# Assessment of the potential of novel and classical opioids to induce respiratory depression in mice

**DOI:** 10.1111/bph.16199

**Published:** 2023-08-22

**Authors:** Rob Hill, Julie Sanchez, Laura Lemel, Mirjana Antonijevic, Yselkla Hosking, Shailesh N. Mistry, Andrew C. Kruegel, Jonathan A. Javitch, J. Robert Lane, Meritxell Canals

**Affiliations:** ^1^ Division of Physiology, Pharmacology and Neuroscience, School of Life Sciences, Queen's Medical Centre University of Nottingham Nottingham UK; ^2^ Centre of Membrane Proteins and Receptors, Universities of Nottingham and Birmingham Midlands UK; ^3^ Division of Biomolecular Science and Medicinal Chemistry, School of Pharmacy, University of Nottingham Biodiscovery Institute University Park Nottingham UK; ^4^ Department of Chemistry Columbia University New York New York USA; ^5^ Departments of Psychiatry and Molecular Pharmacology and Therapeutics Columbia University Vagelos College of Physicians & Surgeons New York New York USA; ^6^ Division of Molecular Therapeutics New York State Psychiatric Institute New York New York USA

**Keywords:** anti‐nociception, opioid, opioid receptor, respiratory depression, therapeutic window

## Abstract

**Background and Purpose:**

Opioid‐induced respiratory depression limits the use of μ‐opioid receptor agonists in clinical settings and is the main cause of opioid overdose fatalities. The relative potential of different opioid agonists to induce respiratory depression at doses exceeding those producing analgesia is understudied despite its relevance to assessments of opioid safety. Here we evaluated the respiratory depressant and anti‐nociceptive effects of three novel opioids and relate these measurements to their in vitro efficacy.

**Experimental Approach:**

Respiration was measured in awake, freely moving male CD‐1 mice using whole body plethysmography. Anti‐nociception was measured using the hot plate test. Morphine, oliceridine and tianeptine were administered intraperitoneally, whereas methadone, oxycodone and SR‐17018 were administered orally. Receptor activation and arrestin‐3 recruitment were measured in HEK293 cells using BRET assays.

**Key Results:**

Across the dose ranges examined, all opioids studied depressed respiration in a dose‐dependent manner, with similar effects at the highest doses, and with tianeptine and oliceridine showing reduced duration of effect, when compared with morphine, oxycodone, methadone and SR‐17018. When administered at doses that induced similar respiratory depression, all opioids induced similar anti‐nociception, with tianeptine and oliceridine again showing reduced duration of effect. These data were consistent with the in vitro agonist activity of the tested compounds.

**Conclusion and Implications:**

In addition to providing effective anti‐nociception, the novel opioids, oliceridine, tianeptine and SR‐17018 depress respiration in male mice. However, the different potencies and kinetics of effect between these novel opioids may be relevant to their therapeutic application in different clinical settings.

**Abbreviations**
AUC: area under the curve
BRET: bioluminescence resonance energy transfer
DAMGO: D‐Ala^2^, N‐MePhe^4^, Gly‐ol‐enkephalin
DMEM: Dulbecco's modified Eagle's medium
HEK cells: human embryonic kidney cells
HPLC: high‐performance liquid chromatography
MPE: maximum possible effect
MV: minute volume
RLuc: *Renilla* luciferase

**What is already known?**

- Tianeptine, oliceridine and SR‐17018 are novel mu opioid receptor agonists.
- Opioid‐induced respiratory depression (OIRD) is usually assessed at doses that produce anti‐nociception.

**What does this study add?**

- Tianeptine, oliceridine and SR‐17018 induce respiratory depression with different duration of effect.

**What is the clinical significance?**

- Pre‐clinical assessment of OIRD at doses beyond those producing effective anti‐nociception is relevant for opioid‐safety.

## 1 INTRODUCTION

Agonists of the μ‐opioid receptor, such as morphine, are the mainstay treatment for acute severe pain as well as chronic cancer pain. However, opioids induce significant adverse effects including respiratory depression, constipation, hyperalgesia, dependence and addiction. Respiratory depression is the primary cause of death in the misuse of both illicit and prescription opioids. In 2020, fatal opioid overdoses surpassed 100,000 in the USA (Dyer, 2021) and both the number and rate of increase in overdose deaths were the highest in the United Kingdom since records began (ONS, 2019). For this reason, intensive research has focused on the development of opioid agonists that retain analgesic efficacy yet have a reduced side effect profile, to enhance clinical safety and reduce opioid‐related deaths.

Both the desired analgesic effect and undesired side effects of opioids are mediated through activation of the μ‐receptor (Matthes et al., 1996), a G_i/o/z_ G protein‐coupled receptor (GPCR). While analgesia is understood to be mediated by G protein signalling at the μ‐receptor, the cellular mechanisms responsible for the induction of opioid side effects have been the subject of debate. Data from arrestin‐3 knock‐out mice showing reduced respiratory depression in response to morphine (Raehal et al., 2005), suggested that arrestin‐3 may mediate this deleterious effect. This stimulated drug discovery efforts toward the identification of biased μ‐receptor agonists that preferentially activate G protein signalling over arrestin pathways, which were hypothesised to retain anti‐nociceptive properties but display reduced respiratory depression (Bohn, 2017; Dekan et al., 2019; Dewire et al., 2013; Groom et al., 2020; Kelly, 2013; Luttrell, 2014; Manglik et al., 2016; Schmid et al., 2017; Váradi et al., 2016; Violin et al., 2014; Zheng et al., 2010). However, more recent evidence suggests that arrestin‐3 does not mediate opioid‐induced respiratory depression (OIRD) (Azevedo Neto et al., 2020; Bachmutsky et al., 2021; He et al., 2021; Kliewer et al., 2019, 2020). Nevertheless, some of the compounds initially identified as G protein‐biased μ‐receptor agonists appear to have improved therapeutic profiles (Brzezinski et al., 2021). These observations may be reconciled by recent studies suggesting that the low intrinsic efficacy of these opioids may underlie such wider separation, or therapeutic window, between their anti‐nociceptive and respiratory depressant doses (Azevedo Neto et al., 2020; Benredjem et al., 2019; Conibear & Kelly, 2019; Gillis, Gondin, et al., 2020; Pineyro & Nagi, 2021). The low‐efficacy μ‐receptor agonist buprenorphine has been implicated in lethal overdose much less frequently than other opioids (Fatseas & Auriacombe, 2007); while clinical trials suggest that its partial agonism did not appear to limit its clinical analgesic effect (Pergolizzi et al., 2010; Raffa et al., 2014), its low intrinsic efficacy is assumed to underlie its plateau of effect on human respiration. A confounding factor in the study of buprenorphine, in addition to its lack of selectivity (Kress, 2009), is the production of active metabolites such as norbuprenorphine, which has lower affinity but higher efficacy than the parent compound (Huang et al., 2001). This metabolite has been implicated in preclinical studies of buprenorphine respiratory depression and, in the rare cases of mortality, has been detected in blood plasma in amounts similar to that of buprenorphine (Kim et al., 2012).

Measurements of anti‐nociception are often the first in vivo assessment for opioid action, but they require set temperature and latency time cut‐offs due to ethical considerations in preventing animal suffering. Thus, differences in anti‐nociceptive efficacy will not be detected beyond this ceiling. The level of this threshold can vary between different studies and may affect the observed anti‐nociceptive potency of an opioid. Importantly, increased noxious stimulus intensities have not only been associated with decreased latencies but also with reduced anti‐nociceptive efficacy (Zimet et al., 1986). Therefore, experimental conditions can affect not just potency but also potential observed differences in maximal efficacy (Hill & Canals, 2022). These anti‐nociception measurements often inform the doses tested in subsequent measurements of respiratory depression, particularly when the aim of the study is to identify agonists that display a dose‐dependent separation between these two effects. However, while estimation of the therapeutic window between anti‐nociceptive and respiratory depressant effects may be a useful indicator of relative opioid safety, it is also important to compare the ability of different opioids to cause respiratory depression at higher doses that might reflect instances of abuse and overdose.

Here, we evaluated the respiratory depressant effects of three novel opioid agonists; oliceridine, tianeptine and SR‐17018. Oliceridine, originally identified as a μ‐receptor G protein‐biased agonist (Dewire et al., 2013), has received FDA approval for the management of moderate to severe pain in controlled clinical settings in adults for whom alternative treatments are inadequate (Lambert & Calo, 2020). SR‐17018 has been identified as a G protein‐biased agonist that provides effective anti‐nociception without significant respiratory depression (Schmid et al., 2017). Recent reports have suggested that SR‐17018 acts as a non‐competitive agonist at the μ‐receptor (Stahl et al., 2021), although the recent cryo‐EM structure determinations and molecular modelling of the μ‐receptor bound to SR‐17018 show the ligand occupying the classical orthosteric site also occupied by morphine and fentanyl (Zhuang et al., 2022). Oliceridine and SR‐17018 have been reported to have lower relative efficacies than morphine. Finally, tianeptine is a low affinity μ‐receptor agonist, clinically used as an antidepressant at low doses (Gassaway et al., 2014; Samuels et al., 2017). We compared these three novel μ‐receptor agonists with several clinically used opioids. Our results show that all tested opioid agonists induce dose‐dependent respiratory depression with a similar effect at the highest dose tested although with different potencies and kinetics of effect. When tested at doses that produced equal respiratory depression, all opioids induced equivalent anti‐nociception.

## 2 METHODS

### 2.1 Animals

All animal care and experimental procedures complied with the UK Animals (Scientific Procedures) Act 1986, the European Communities Council Directive (2010/63/EU), and were approved by the Ethical Review Board at the University of Nottingham (project licence P3C7EE0BA). Animal studies are reported in compliance with the ARRIVE guidelines (Percie du Sert et al., 2020) and with the recommendations made by the *British Journal of Pharmacology* (Lilley et al., 2020).

Male CD‐1 mice (Charles River, UK) weighing approximately 25–35 g (over the lifetime of the experiment) were group housed, three per cage, in an environment maintained at 22°C, on a reversed 12‐h/12‐h dark–light cycle with food and water available ad libitum.

### 2.2 Experimental design

Data from previous experiments (Hill et al., 2016; Hill, Dewey, et al., 2018) where respiratory depression or anti‐nociception were measured following acute opioid administration in naïve mice were used to guide a priori power analyses using G*Power (version 3.1.9). These calculations indicated that n = 6 (respiration experiments) or n = 10 (anti‐nociception experiments) for each group would produce a significant result if an actual effect occurred. Experiments were performed in the dark (active) phase. The experimenter was blinded to all drug treatments, both during the experiment and data analysis. Cages of mice were randomly allocated to drug treatments, such that all mice in one cage received the same drug to minimise social conflict between different drug effects. All mice within a single cage were experimented on at distinct times and were also randomly assigned to separate plethysmography chambers such that treatment was by mouse not cage. A total of 370 mice were used in the study, with no mouse being used more than once.

### 2.3 Drug administration

[D‐Ala^2^, *N*‐MePhe^4^, Gly‐ol]‐enkephalin (DAMGO), morphine hydrochloride, methadone hydrochloride, oxycodone hydrochloride, tianeptine sodium salt, oliceridine hydrochloride and SR‐17018 were used in this investigation.

For in vivo experiments morphine hydrochloride and tianeptine sodium salt were dissolved in saline. Oliceridine hydrochloride was initially dissolved in DMSO with dilution to final concentrations in saline. DMSO concentrations did not exceed 4% for the highest dose of oliceridine. These opioids were all administered by i.p. injection.

For use in vivo, SR‐17018 was dissolved in DMSO and Tween80 before dilution to final concentrations in distilled water (Schmid et al., 2017). The most concentrated vehicle was DMSO/Tween80/distilled water in a 4:16:80 ratio. SR‐17018 was administered orally as described by Grim et al. (2020). Oxycodone and methadone were used as orally dosed comparators for SR‐17018 (as in Grim et al., 2020) and dissolved in the same vehicle to ensure that changes in the rate of absorption induced by vehicle density were consistent across these drugs. All drugs were administered in 0.1‐ml volumes.

### 2.4 Mouse respiration

Respiration was measured in freely moving mice using plethysmography chambers (EMKA Technologies, Paris, France) supplied with room air or a 5% CO_2_ in air mixture (Figure S5) as described previously (Hill et al., 2016). Mice were habituated to respiratory chambers for 30 min the day prior to experimentation. Respiratory parameters were recorded (IOX software—EMKA Technologies, Paris, France) and averaged over 5‐min periods. On the day of the experiment, respiration was measured for 20 min prior to drug administration, of which the last 10 min were taken as the baseline.

Data are presented as percentage change from the pre‐drug minute volume baseline, calculated from data for each individual mouse before data being collated and plotted as a mean. Data are presented as a percentage change from each mouse's pre‐drug baseline controls to account for variations in individual minute volume due to variations in mouse size. Raw minute volume data are presented in Figure S1.

### 2.5 Calculation of equi‐effective doses in respiratory depression

The maximum depression of respiration induced at any time point for a given dose was determined for each drug and each mouse. These data were fitted to linear and polynomial regressions. Linear regressions were found to produce the best fit for each agonist (Figure S2). Slope equations were then used with a set *Y*‐value of 40% (the degree of respiratory depression) to determine the log dose value predicted to induce this level of effect.

### 2.6 Anti‐nociception

Mice were habituated to the hot plate for 30 min with the hot plate inactive on the day prior to experimentation. For experimentation, mice were placed on a hot plate at 52.5°C and the latency to exhibit a pain‐like response (defined as paw withdrawal, jumping, paw licking and fluttering of the hind limbs) was measured. A maximum cut‐off time of 20 s was used to prevent tissue damage, and measurements were taken 15 min apart. Data are presented as the percentage of maximum possible effect (%MPE), which was calculated as: %MPE = [(test latency − control latency)/(20 − control latency)] × 100 were control latency was the average of the three baseline reads. Raw latency data are presented in Figure S3.

### 2.7 BRET experiments

HEK 293 cells (CLS Cat# 300192/p777_HEK293, RRID:CVCL_0045) were cultured at 37°C in Dulbecco's modified Eagle's medium (DMEM) supplemented with 10% foetal bovine serum. Cells were seeded onto 10‐cm dishes and grown to 70% confluency before transfection. Cells were transiently transfected with 1 μg of μ‐receptor‐RLuc8 and 4 μg of Nb33‐Venus, or 4 μg of arrestin‐3‐Venus and 2 μg of GRK2 (all constructs described previously in Gillis, Gondin, et al., 2020). Twenty‐four hours after transfection cells were detached and re‐plated in media in a white bottom Poly‐D‐Lys coated 96‐well plate. Forty‐eight hours after transfection, cells were incubated with phosphate‐buffered saline (PBS) for 30 min, 10 μl of coelenterazine h (final concentration of 5 μM) was added to each well, and baseline BRET was measured for 4 min. Serial dilutions were freshly prepared for each opioid agonist from stock solutions at 10 mM (compounds dissolved in distilled water—morphine, DAMGO, tianeptine and oliceridine—or DMSO—oxycodone, methadone and SR‐17018). These serial dilutions were then added to the cells for a final concentration range of 10–0.01 nM (DMSO concentration did not exceed 0.1% in any given well unless otherwise stated in Figure S6). BRET was measured for 30 min using a PHERAstar FSX plate reader (BMG LABTECH, Ortenberg, Germany) and the BRET 1 plus filter set (acceptor, 535 ± 30 nm, and donor, 475 ± 30 nm). BRET ratio was determined as the fluorescence intensity of the Venus acceptor over the luminescence emitted by the RLuc8 donor. Nb33 or arrestin‐3 recruitment concentration response curves show the 10 min response after opioid addition normalised to the response elicited by DAMGO.

The results of concentration‐response experiments were analysed using Prism 9.3 (GraphPad Software Inc., San Diego, CA) and fitted using the following three parameter equation:

(1) $$\text{response}=\text{bottom}+\frac{\mathrm{top}-\text{bottom}}{1+10^{\left(\log\;{\mathrm{EC}}_{50}-\log\left[A\right]\right)}}$$

where top and bottom represent the maximal and minimal asymptote of the concentration response curve, [*A*] is the molar concentration of agonist and EC_50_ is the molar concentration of agonist required to give a response half‐way between maximal and minimal asymptote. Data show the mean ± SEM of at least five independent experiments performed in duplicate. Statistical significance was determined using one‐way ANOVA with Tukey's‐comparison.

### 2.8 SR‐17018 mesylate salt formation

#### 2.8.1 General chemistry

All commercially available compounds were used as received without further purification. Other reagents were used as provided by chemical companies. NMR spectra were recorded on a Bruker‐AV 400 MHz Spectrometer (400.13 MHz for ^1^H) in MeOD‐*d*_*4*_. Data are reported as follows: chemical shifts (*δ*) reported in parts per million (ppm), relative to tetramethylsilane (TMS) as internal standard and recorded. The following abbreviations are used to describe multiplicity: s, singlet; d, doublet; t, triplet; q, quartet; m, multiplet; coupling constants (*J*) reported in hertz (Hz), and integration.

#### 2.8.2 1‐(4‐Chlorobenzyl)‐4‐(5,6‐dichloro‐2‐oxo‐2,3‐dihydro‐1H‐benzo[d]imidazol‐1‐yl)piperidin‐1‐ium methanesulfonate (SR‐17018 mesylate salt)

SR‐17018 free base (20 mg, 48.7 μmol) was dispersed in ethanol (12.5 ml·mmol^−1^). Methanesulfonic acid (3.16 μl, 48.7 μmol, 1 eq) was added and the mixture was stirred at 60°C for 30 min. Solvent was evaporated under reduced pressure and the remaining residue was dissolved in water (1.5 ml) and MeCN (0.5 ml), frozen and lyophilized overnight to afford 1‐(4‐chlorobenzyl)‐4‐(5,6‐dichloro‐2‐oxo‐2,3‐dihydro‐1*H*‐benzo[*d*]imidazol‐1‐yl)piperidin‐1‐ium methanesulfonate (16.8 mg, 33.1 μmol, 68% yield) as a white powder. ^1^H NMR (400 MHz, MeOD‐*d*_*4*_) δ 7.59–7.52 (m, 4H), 7.46 (s, 1H), 7.20 (s, 1H), 4.56–4.46 (m, 1H), 4.38 (s, 2H), 3.65 (d, *J* = 12.5 Hz, 2H), 3.24 (t, *J* = 13.1 Hz, 2H), 2.80–2.68 (m, 5H), 2.09 (d, *J* = 14.2 Hz, 2H). Spectral and analytical data matched with literature (Schmid et al., 2017).

### 2.9 SR‐17018 solubility tests

#### 2.9.1 Sample preparation

Stock solutions (10.0 ml) of DMSO/PBS (1:99), DMSO/PBS (10:90) and DMSO/Tween 80/MilliQ water (4:16:80) were freshly prepared at ambient temperature. Both SR‐17018 free base (Adooq, USA) and SR‐17018 mesylate salt (as prepared above) underwent solubility testing in each solvent system. For each solvent system, 3 × 2‐ml amber sample vials were prepared, each containing approximately 1 mg of either SR‐17018 free base or SR‐17018 mesylate salt and 500 μl of the appropriate solvent system. The vials were sonicated for 1 min before visual inspection. All vials were found to contain a white suspension indicating an excess of SR‐17018 or SR‐17018 mesylate salt was present. All the samples were placed in a pre‐heated aluminium heating block and stirred on a stirrer hotplate at 37°C for 89 hours. Each sample was then centrifuged at 15115 × *g* for 5 min. The supernatant from each sample was then filtered through a syringe filter (Merck Millex‐HA mixed cellulose esters 0.45‐μm pore size, 33‐mm diameter), before diluting a 50‐μl aliquot to 500 μl (1 in 10 dilution) with MeOH. The concentration of each sample was determined following HPLC analysis on two separate systems and reference to the standard calibration curve as described below.

#### 2.9.2 Calibration curve preparation

SR‐17018 (1.05 mg, 2.56 μmol) was dissolved in DMSO/MeOH (1:1, 1.0 ml). This stock solution then underwent 11 serial dilutions (1 in 2) to give calibration curve samples (concentration range 0.61–1241.72 μM). Standard calibration curves were generated following LCMS analysis as described below.

#### 2.9.3 LCMS analyses

Shimadzu UFLCXR HPLC system coupled to an Applied Biosystems MDS SCIEX API2000 electrospray ionisation mass spectrometer. Samples were eluted through a Phenomenex Gemini‐NX C18 110A column (2 mm × 50 mm × 3 μm) using a gradient method (5% solvent B to 95% solvent B over 5 min at a flow rate 0.5 ml·min^−1^) with UV detection at 254 nm. The calibration curve was found to be linear in the range 0.25–255.00 μg·ml^−1^ (0.61–620.86 μM). Figure S7 and Table S4 show full calibration curve and experimental solubility data.

### 2.10 Data and statistical analysis

The data and statistical analyses comply with the recommendations on experimental design and analysis in pharmacology (Curtis et al. 2022). All data are displayed as means ± SEM. Comparison between the effects of different drugs were analysed using one‐way ANOVA, with appropriate post‐test comparison between groups (as indicated in the corresponding figure legends). Statistical comparisons across time or in a 2 × 2 factorial were made using a two‐way ANOVA, with Tukey's post‐comparison test used for between group analysis. Statistical significance and F (dfn, dfd) values, are given in Table S2, with column and row factors indicated for two‐way ANOVA analyses. GraphPad Prism 8 was used for all statistical analyses.

### 2.11 Materials

DAMGO was supplied by Tocris Bioscience (Avonmouth, UK), morphine hydrochloride by Macfarlan Smith (Edinburgh, UK) and was a kind gift from Graeme Henderson Bristol, UK). Methadone hydrochloride and oxycodone hydrochloride were supplied by Sigma Aldrich, (Gillingham, Dorset, UK and tianeptine sodium salt, oliceridine hydrochloride and SR‐17018 by Adooq (Irvine, CA, USA).

### 2.12 Nomenclature of targets and ligands

Key protein targets and ligands in this article are hyperlinked to corresponding entries in https://www.guidetopharmacology.org/ and are permanently archived in the Concise Guide to PHARMACOLOGY 2021/2022 (Alexander et al., 2021).

## 3 RESULTS

### 3.1 Depression of respiration by acute administration of opioid agonists

The μ‐receptor agonists were divided in two groups based on their administration routes. Oliceridine and tianeptine were administered intraperitoneally (i.p.), as previously described (Dewire et al., 2013; Gassaway et al., 2014; Hill, Disney, et al., 2018a; Samuels et al., 2017) and i.p. morphine was used as a comparator agonist (Hill, Dewey, et al., 2018). In contrast, SR‐17018 has been recently reported to have significantly improved bioavailability upon oral administration compared to i.p. (Grim et al., 2020). As the effects of SR‐17018 on respiration have previously been measured only following i.p. administration, this compound together with the comparators oxycodone and methadone were tested here when administered orally.

**FIGURE 1:**
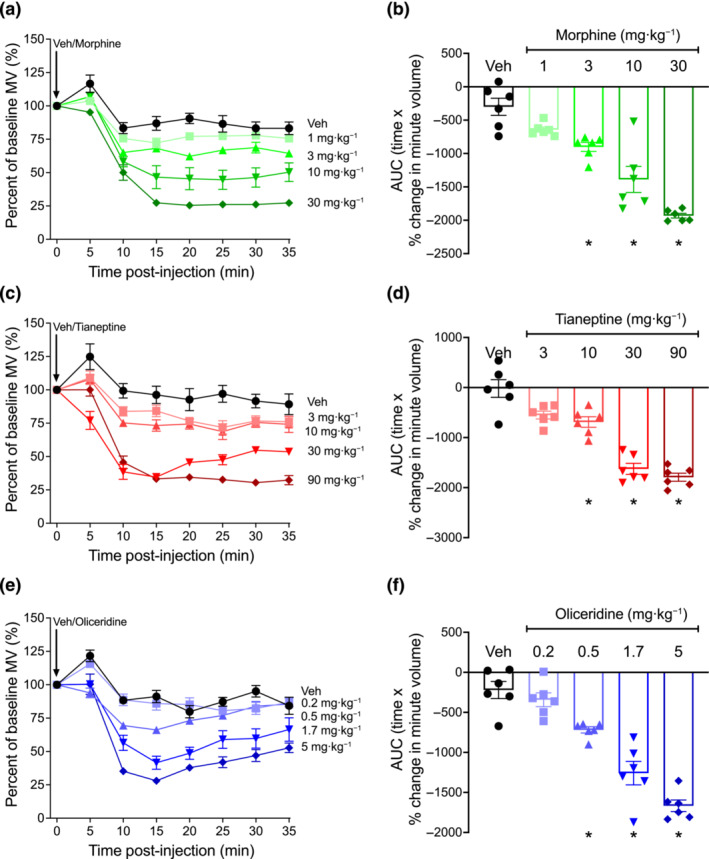
Respiratory depression induced by morphine, tianeptine and oliceridine in mice. (a,b) Morphine (1–30 mg·kg^−1^ i.p.), (c,d) tianeptine (3–90 mg·kg^−1^ i.p.) and (e,f) oliceridine (0.2–5 mg·kg^−1^ i.p.) dose‐dependently depressed mouse respiration. Data shown (a, c, e) are means ±SEM or (b, d, f) individual values with means ±SEM; n = 6 for all groups. Comparison of area under the curve (AUC) values in (b) (*F* = 2.93; DFn = 4; DFd = 25), (d) (*F* = 0.74; DFn = 4; DFd = 25) and (f) (*F* = 1.29; DFn = 4; DFd = 25) were made by one‐way ANOVA, with Tukey's comparison. *P < 0.05, significantly different from vehicle (Veh) control. Statistical test details are provided in Table S2.

The respiratory depressant effects of opioid agonists were assessed in mice breathing room air within a whole‐body plethysmograph. Following i.p. administration, morphine (1–30 mg·kg^−1^), tianeptine (3–90 mg·kg^−1^) and oliceridine (0.2–5 mg·kg^−1^) dose‐dependently depressed respiration as shown by a decrease of the minute volume from the baseline over a 35 min observation period (Figures 1 and S1). Over this 35‐min period, the respiratory depression induced by oliceridine and tianeptine (up to 30 mg·kg^−1^) showed a tendency to return to baseline levels (Figure 1c,e) compared to the sustained respiratory depression induced by all doses of morphine (Figure 1a). The kinetics of these effects are in agreement with the pharmacokinetics and clearance rates that have been described for these three opioids (Altarifi et al., 2017; Dewire et al., 2013; Gassaway et al., 2014; Samuels et al., 2017; Soergel et al., 2014).

**FIGURE 2:**
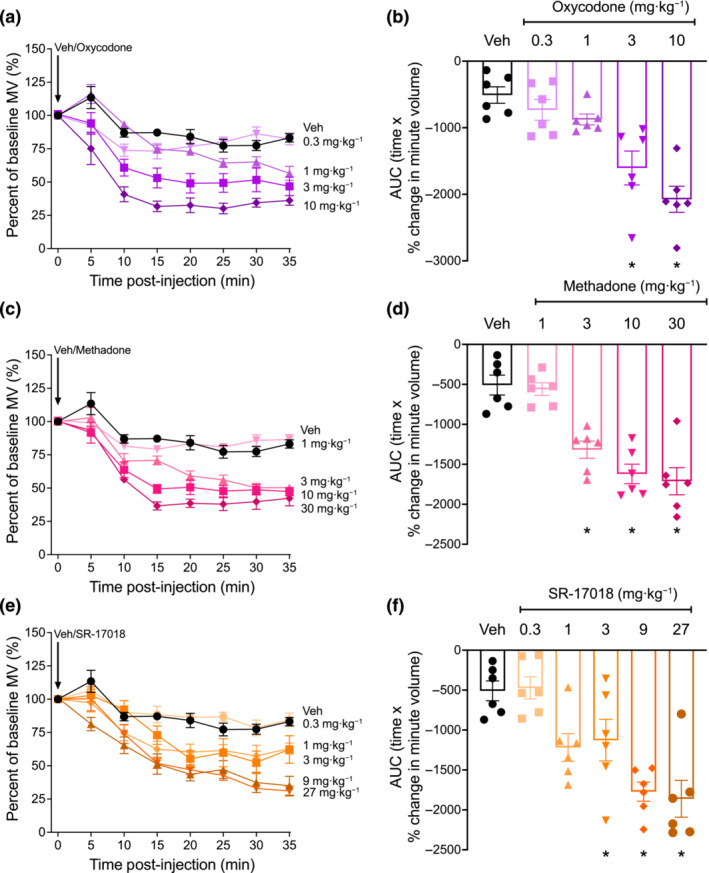
Respiratory depression induced by oxycodone, methadone and SR‐17018 in mice. (a,b) oxycodone (0.3‐ to 10‐mg·kg^−1^ oral gavage), (c,d) methadone (1‐ to 30‐mg·kg^−1^ oral gavage) and (e,f) SR‐17018 (0.3‐ to 27‐mg·kg^−1^ oral gavage) dose‐dependently depressed mouse respiration. Data shown (a, c, e) are means ±SEM or (b, d, f) individual values with means ±SEM; n = 6 for all groups. Comparison of area under the curve (AUC) values in (b) (*F* = 0.68; DFn = 5; DFd = 30), (d) (*F* = 1.71; DFn = 4; DFd = 25) and (f) (F = 0.47; DFn = 4; DFd = 25) were made by one‐way ANOVA using Tukey's comparison. *P < 0.05, significantly different from vehicle (Veh) control. n = 6 for all groups. Statistical test details are provided in Table S2.

Following oral administration, oxycodone (0.3–10 mg·kg^−1^), methadone (1–30 mg·kg^−1^) and SR‐17018 (0.3–27 mg·kg^−1^) dose‐dependently decreased respiration with a sustained effect for the 35‐min observation period at doses above 0.3 mg·kg^−1^ (Figures 2 and S1).

Thus, all tested opioids were found to depress respiration and to induce a similar effect at the highest doses (Figures 1 and 2 and Table S1). However, the potency and the kinetics of effect produced by each opioid (reflected in their traces and AUC) suggest a unique profile for each drug.

### 3.2 Calculation and assessment of equi‐effective respiratory depressant doses

**FIGURE 3:**
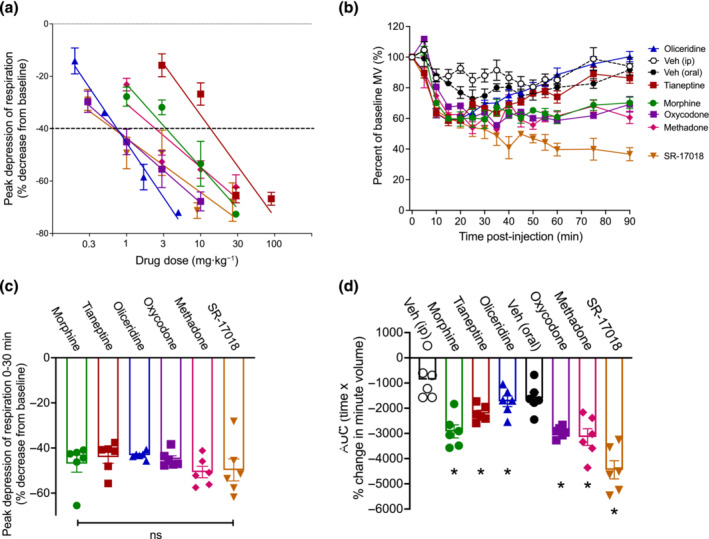
Equi‐effective doses of opioids and induction of respiratory depression in mice. (a) the max induced effect (<35 min) for each opioid was plotted againstdose and linear regression performed to estimate equi‐effective doses. (b) Estimated equi‐effective doses for morphine (3.44 mg·kg^−1^), tianeptine (12.74 mg·kg^−1^), oliceridine (0.745 mg·kg^−1^), oxycodone (0.71 mg·kg^−1^), methadone (2.5 mg·kg^−1^) and SR‐17018 (0.66 mg·kg^−1^) depressed respiration with no significant difference in maximum respiratory depression in the first 30 min. The effect of tianeptine and oliceridine was not significantly different from vehicle after 60 min; two‐way ANOVA with Tukey's comparison. (c) Peak effect and (d) area under the curve (AUC) analysis for each opioid. Data shown (a, b) are means ±SEM or (c, d) individual values with means ±SEM; n = 6 for all groups. *P < 0.05, significantly different from vehicle (Veh) control; ns = non‐significant; (c, d) one‐way ANOVA with Tukey's comparison. Statistical test details are provided in Table S2.

The maximum respiratory depressant effect induced by each drug at each dose in each mouse (Table S1) was used to estimate an equi‐effective dose of each opioid that produced ~40% respiratory depression (Figure 3a); morphine (3.44 mg·kg^−1^), tianeptine (12.74 mg·kg^−1^), oliceridine (0.745 mg·kg^−1^), oxycodone (0.71 mg·kg^−1^), methadone (2.5 mg·kg^−1^) and SR‐17018 (0.66 mg·kg^−1^). Of note, the equi‐effective concentrations determined when AUC values were plotted for each drug dose were similar to those obtained when using the maximum observed effect measures (Figure S2).

The estimated equi‐effective doses were then administered to mice (i.p. or orally) and respiration measured for 90‐min post‐administration to capture the kinetic profile of the respiratory depressant effect of these drugs beyond the initial 30 min (Figure 3b).

As expected, based on the use of equi‐effective doses, the effects of morphine, tianeptine, oliceridine, oxycodone, methadone and SR‐17018 within the first 30 min of observation were not significantly different (Figures 3c and S3 and Table S2). However, morphine, methadone and oxycodone induced more prolonged respiratory depression than tianeptine and oliceridine, as the effect of these latter two opioids was not significantly different from vehicle levels of respiration 60 min after administration (Figure 3b). During the same 60‐ to 90‐min period, SR‐17018 continued to induce maximum levels of respiratory depression (Figure 3b). These results are in agreement with the previously reported pharmacokinetics of these drugs (Altarifi et al., 2017; Dewire et al., 2013; Gassaway et al., 2014; Samuels et al., 2017; Soergel et al., 2014). The observation of pronounced respiratory depression upon oral administration of SR‐17018 is different from the minimal respiratory depression observed when SR‐17018 was administered i.p. (Gillis, Gondin, et al., 2020; Schmid et al., 2017) and suggests that the route of administration of this compound can affect its respiratory depressant effects. Indeed, when we administered SR‐17018 i.p. (0.66 mg·kg^−1^), no significant respiratory depression or anti‐nociception were observed (Figure S4). Equi‐effective doses of all opioids also induced the same level of effect on respiration when tested in mice breathing a 5% CO_2_ in air mixture (Figure S5).

### 3.3 Anti‐nociceptive assessment of equi‐effective respiratory depressant doses

**FIGURE 4:**
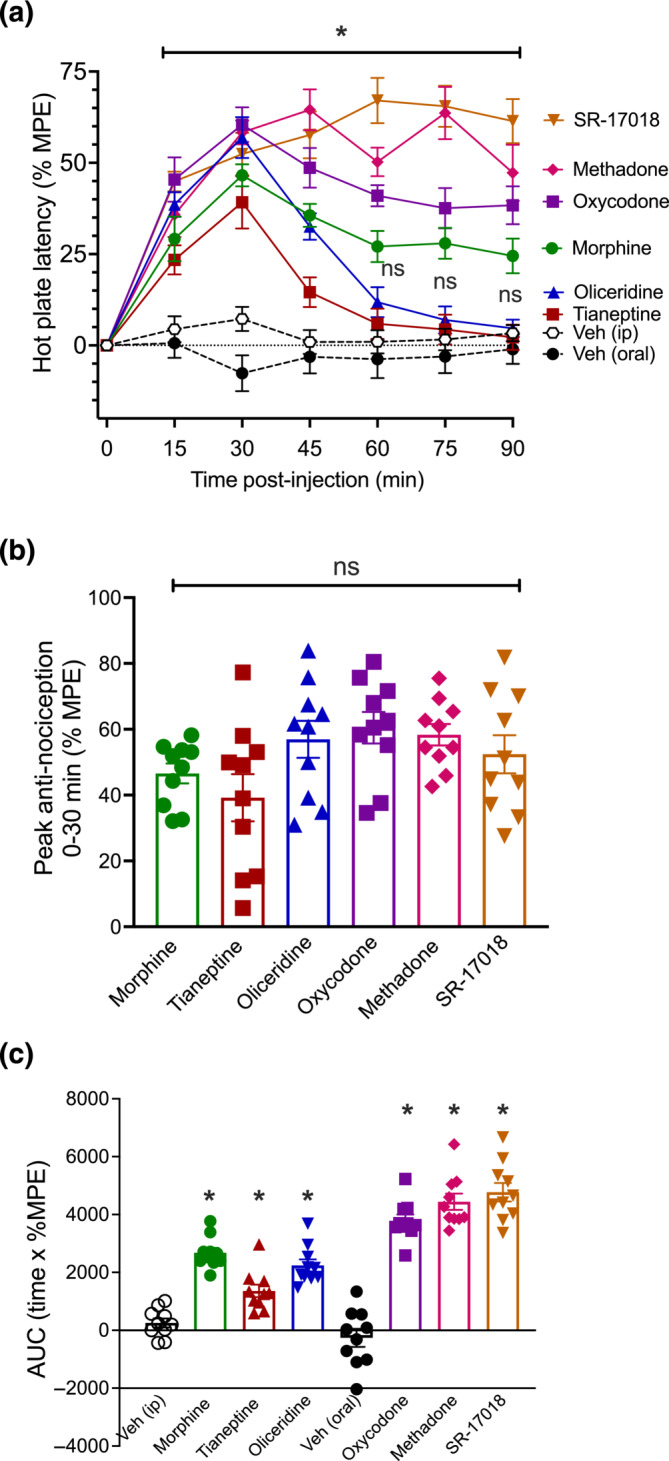
Equi‐effective respiratory doses of opioids and their induction of anti‐nociception in mice. (a) Morphine (3.44 mg·kg^−1^), tianeptine (12.74 mg·kg^−1^), oliceridine (0.745 mg·kg^−1^), oxycodone (0.71 mg·kg^−1^), methadone (2.5 mg·kg^−1^) and SR‐17018 (0.66 mg·kg^−1^) all induced significant anti‐nociception. Data shown are means ±SEM. n = 10 for all groups. *P < 0.05, significantly different as indicated. The effect of tianeptine and oliceridine was not significantly different from vehicle after 60 min (ns); two‐way ANOVA with Tukey's comparison. (b) No statistical difference was detected at 30‐min post‐injection across the six opioids. (c) Area under the curve (AUC) analysis of data in (a) showed induction of anti‐nociception by all opioids, relative to their corresponding vehicle. In (b, c), data shown are individual values with means ±SEM; n = 10 for all groups. *P < 0.05, significantly different from vehicle (Veh) control; ns indicates non‐significant; one‐way ANOVA with Tukey's comparison. Statistical test details are provided in Table S2.

Equi‐effective doses estimated from the respiratory depression data were then administered to measure anti‐nociception in the hot plate assay for 90‐min post‐opioid administration (Figure 4a). All opioids induced significant anti‐nociception and the degree of anti‐nociception induced at 30 min was not significantly different across opioids (Figure 4b,c). However, oliceridine and tianeptine displayed anti‐nociceptive kinetics comparable to those seen in the respiratory depression assay and as previously described (Altarifi et al., 2017, Dewire et al., 2013, Gassaway et al., 2014, Samuels et al., 2017, Soergel et al., 2014), with the effect of both opioids returning to baseline levels 60‐min post‐administration. In contrast, the anti‐nociception induced by morphine, oxycodone, methadone and SR‐17018, was maintained for the entire period of observation.

### 3.4 Measurement of Nb33 and arrestin recruitment to the μ‐receptor

**FIGURE 5:**
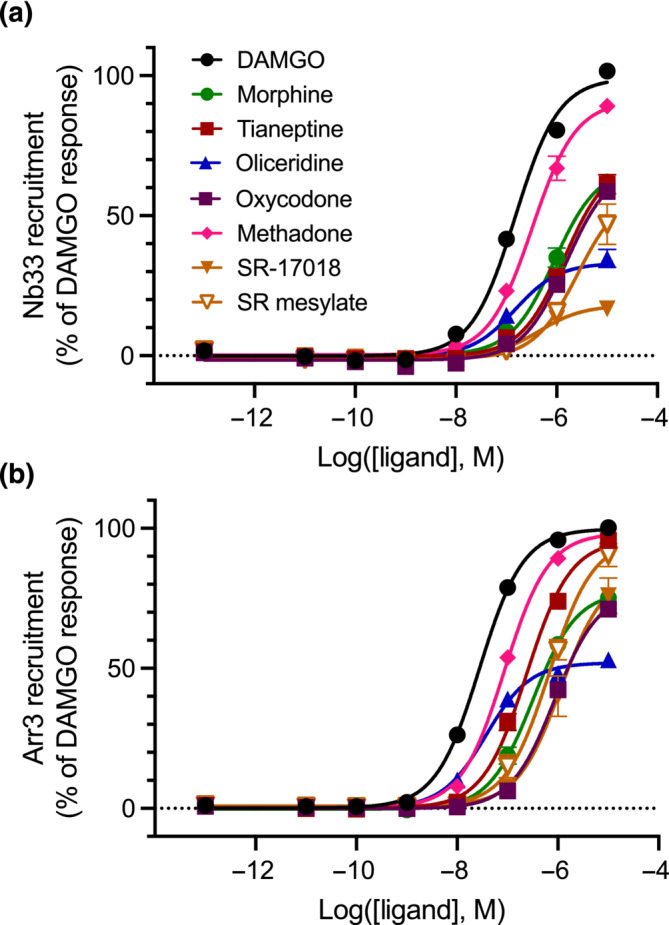
Opioid‐induced recruitment of Nb33 and arrestin‐3 to μ‐receptors in HEK 293 cells. Concentration response‐induced recruitment of the conformationally selective nanobody Nb33‐Venus (a) and of arrestin‐3‐Venus in the presence of GRK2 (b) to the μ‐receptor‐RLuc8. Data shown are means ± SEM; n = 5 experiments performed in duplicate.

To complement the studies above we assessed the in vitro pharmacology of the six opioid agonists by monitoring the recruitment of the conformationally selective nanobody, Nb33 and of arrestin‐3 to the μ‐receptor in HEK 293 cells. DAMGO was used as a reference ligand (Figure 5). The order of potencies and efficacies observed in these assays agreed with previously described results (Table S3) (Gillis, Gondin, et al., 2020). The only exception was SR‐17018, which showed higher efficacy than we had previously determined, although in agreement with more recent reports (Mösslein et al., 2022). Moreover, we note that the original reports describing this compound used the mesylate salt of SR‐17018. In our hands, the SR‐17018 mesylate salt showed increased efficacy in both assays compared to the free base. The known poor solubility of this compound (Grim et al., 2020; Schmid et al., 2017; Stahl et al., 2021) may have affected the determinations of in vitro efficacy of SR‐17018 in live cells, where vehicles for administration containing >1% DMSO or any detergent (such as Tween 80, used in vivo) significantly impair cell viability (Figure S6).

We thus investigated differences in compound saturating solubility in the different vehicles (1% DMSO in vitro, DMSO/Tween 80/Water (4:16:80) in vivo). Briefly, the saturating solubility of SR‐17018 and its corresponding mesylate salt in PBS/DMSO (1:99 and 10:90) and DMSO/Tween 80/Water (4:16:80) was assessed via quantitative LCMS analysis (see Section 2). Where possible, the mean saturating solubility across three individual samples was determined in each solvent system. For some solvent conditions, sample solubility was too low to allow consistent detection to take place within the detection limits of the system. Mean concentrations were determined via reference to a standard calibration curve of SR‐17018 in DMSO/MeOH (see Section 2 and Figure S7 and Table S4). This analysis revealed a difference in the solubility of this compound in the vehicles used for in vitro and in vivo assays, as well as differences between the free base and salt forms. In DMSO/Tween 80/water (4:16:80) SR‐17018 free base was found to have saturating solubilities of 417 ± 36 μM, compared to that of the corresponding mesylate salt of 3,374 ± 779 μM. In contrast, in DMSO/PBS (1:99) SR‐17018 free base was found to have a much lower saturating solubility of 15 μM, comparable to 12 ± 4 μM for the mesylate salt form. The effects of DMSO/PBS (10:90) as a solvent system were also evaluated, giving saturating solubilities of 49 ± 17 μM for the free base and 190 ± 161 μM for the mesylate salt form. These measurements indicate that SR‐17018 free base can reach concentrations that are ~27 times higher in DMSO/Tween 80/water (4:16:80) compared to DMSO/PBS (1:99) (Table S4) and ~274 times higher in the case of the mesylate salt form. As expected, the solubility of the mesylate salt form in DMSO/Tween 80/water (4:16:80) is much higher (~8‐fold) than the parent free base. These observations warrant further investigation. However, the saturating solubility of SR‐17018 in different solvent systems and the differential solubility of the SR‐17018 mesylate salt form may help to explain the variability in results described above and in previous reports. Indeed, the actual concentration present in a particular experiment, particularly above the saturation concentration, would be highly dependent on experiment‐specific factors.

## 4 DISCUSSION

Opioid agonists remain the most effective treatment for acute pain but are limited by the on‐target adverse effects they elicit. Improvement of the separation between the doses that induce pain relief and the doses that induce adverse effects has been the focus for the development of safer opioid analgesics. New drug candidates are typically first evaluated for their ability to induce anti‐nociception in animal models. Their propensity to cause opioid‐induced adverse effects is subsequently assessed at doses where effective anti‐nociception is detected. However, a lack of side effects at anti‐nociceptive doses does not necessarily imply the absence of these effects at higher doses, and the potential of developing side effects has been much less studied. This is particularly important for opioid‐induced respiratory depression (OIRD)—the clinically‐limiting lethal factor in opioid overdose (White & Irvine, 1999)—as the development of tolerance and/or the misuse of these drugs can lead to the administration of doses beyond those that initially produced effective analgesia. Moreover, all anti‐nociceptive measurements in animal models need to set cut offs to avoid tissue damage and animal suffering. Therefore, differences in anti‐nociceptive efficacy cannot be detected beyond a certain threshold and may result in overestimations of a drug's anti‐nociceptive potency and/or efficacy. In contrast, while rodents display resistance to lethality by OIRD, we and others have previously shown that assessment of OIRD using whole body plethysmograpy approaches can differentiate the effects of opioids with different relative efficacies, such as observed with morphine and buprenorphine (Dahan et al., 2005; Gillis, Gondin, et al., 2020; Hill, Disney, et al., 2018c).

The experimental design of the present study was aimed at assessing the potential of μ‐receptor agonists to induce respiratory depression at a range of doses beyond those providing effective anti‐nociception, rather than determining a therapeutic window (i.e., the separation of anti‐nociceptive and respiratory depressant, ED_50_, doses). We compared the action of three novel μ‐receptor agonists: oliceridine, tianeptine and SR‐17018. Oliceridine (Olinvyk, Trevena) has been recently approved by the FDA for the treatment of moderate to severe pain in adults in controlled clinical settings. Originally proposed to be a μ‐receptor G protein‐biased agonist (Dewire et al., 2013), this low‐efficacy μ‐receptor agonist (Gillis, Gondin, et al., 2020) has shown similar respiratory burden and reduced gastrointestinal adverse effects when compared with morphine in clinical trials (Bergese et al., 2019; Singla et al., 2019; Viscusi et al., 2019). Tianeptine is a low affinity μ‐receptor agonist with established clinical efficacy as an antidepressant at low doses (Gassaway et al., 2014; Samuels et al., 2017). While its analgesic, antidepressant, locomotor and hypophagic effects were shown to be mediated by the μ‐receptor (Samuels et al., 2017), the respiratory depressant effects of tianeptine as a μ‐receptor agonist have only been assessed recently (Baird et al., 2022). SR‐17018 was described as a G protein‐biased agonist that acted as an effective analgesic with no significant respiratory depression when administered i.p. (Schmid et al., 2017). A subsequent report revealed that this compound had increased bioavailability when administered orally (Grim et al., 2020) and, to our knowledge, its effects on respiratory depression have not been tested through this route of administration. A recent characterisation of SR‐17018 and its analogues suggests that these compounds may be binding to an allosteric site of the μ‐receptor (Stahl et al., 2021), although recent cryo‐EM structures revealed an orthosteric binding mode (Zhuang et al., 2022). As comparator compounds for these novel opioids, we used reference μ‐receptor agonists that are typically administered through the same route as the test ligands; morphine was used as a reference for the i.p. injected drugs (oliceridine and tianeptine), while oxycodone and methadone were used as references for SR‐17018 via the oral route.

Our data showed that all opioids tested here induced respiratory depression in a dose‐dependent manner with potencies and kinetics that agree with their reported affinities and pharmacokinetics. Measurement of both the maximum observed effect and the kinetics of this effect for each opioid together provides a useful insight into their action. All drugs displayed the same ability to depress respiration at the highest dose tested but differed both in their potency and the duration of this effect. These results contrast with our previous investigations on the respiratory depression induced by the kratom alkaloid mitragynine, which reached a ceiling effect that could be explained, at least in part, by the metabolic saturation of its metabolising enzyme (Hill et al., 2022).

Oliceridine has been shown to have high (~1 nM) affinity for μ‐receptors, rapid brain penetration and fast clearance (Ok et al., 2018), which is reflected in the potency and duration of the respiratory depressant effect induced by this drug. Tianeptine is an opioid agonist that is used clinically to treat depression (Kasper & McEwen, 2008), with a low incidence of poisoning or overdose reports (El Zahran et al., 2018). Tianeptine has lower affinity than oliceridine (~400 nM), but also has rapid brain distribution (with peak levels at 5 min) and rapid clearance through beta‐oxidation (Couet et al., 1990; Royer et al., 1988; Samuels et al., 2017) (undetected in plasma or brain 1 h after administration) consistent with the potency and duration of the respiratory depressant effect observed here. A recent study has investigated the respiratory depressant effects of tianeptine in male Swiss‐Webster mice and showed that at 100 mg·kg^−1^ the effects of tianeptine were sustained (over 80 min), but reversible by the antagonist naloxone (Baird et al., 2022). As discussed below, the kinetics of effect may also contribute to the separation between an effective antidepressant dose and a dose that causes significant respiratory depression. In contrast, and in agreement with our data that revealed a long‐lasting respiratory depressant effect, SR‐17018 has a significantly longer half‐life and has been detected in the brain up to 15‐h post‐administration (24 mg·kg^−1^ twice daily p.o.). When these drugs were tested in anti‐nociceptive assays at doses that produced 40% respiratory depression, they displayed the same profiles over time, with tianeptine and oliceridine returning to baseline levels 60‐min post‐injection and SR‐17018 inducing prolonged anti‐nociception. The relationship between these distinct durations of effect among the various clinically used opioid agonists and their therapeutic efficacy and propensity to cause side effects deserves further investigation.

Our results suggest that an absolute separation of respiratory depression and anti‐nociception through μ‐receptor agonism is unlikely, as all agonists tested here, regardless of their proposed mechanism of action, produced significant respiratory depression at the highest doses tested. Similarly, the unexpectedly low overdose rates of tianeptine, despite its evident ability to depress mouse respiration in the presented data and elsewhere (Baird et al., 2022), may be indicative of the therapeutic separation between the low doses of tianeptine that are effective in the treatment of depression, but remain sub‐threshold for the induction of significant respiratory depression and analgesia. A comparison of tianeptine dose–response curves for the induction of anti‐depressant‐like behaviour and of known opioidergic effects, including respiratory depression, will reveal the effective dose‐separation between therapeutic and adverse effects of this drug in animal models.

The significant respiratory depression induced by SR‐17018 when administered orally was unexpected. Previous data have shown reduced respiratory depression from SR‐17018, when compared with morphine or fentanyl, upon i.p. injection (Gillis, Gondin, et al., 2020; Schmid et al., 2017). While SR‐17018 was shown to be present in the brain at significant concentrations following i.p. injection (Schmid et al., 2017), it also has greatly improved bioavailability when administered orally (Grim et al., 2020). Indeed, our data directly comparing i.p. and oral administration of SR‐17018 suggest that the different route of administration appears to be the most likely explanation for these different results (Figure S4). The generation of an active metabolite responsible for these effects to a greater extent upon oral administration, however, cannot be ruled out.

We and others have previously hypothesised that the low intrinsic efficacy of novel opioid agonists, including SR‐17018, may underlie the greater therapeutic window between anti‐nociception and respiratory depression, when compared with morphine or fentanyl (Gillis, Gondin, et al., 2020; Gillis, Kliewer, et al., 2020). However, as discussed above, when administered orally, SR‐17018 does induce significant respiratory depression. SR‐17018 is known to have limited solubility (Grim et al., 2020; Schmid et al., 2017; Stahl et al., 2021) and the vehicles used for in vivo experiments (containing 10% DMSO and/or detergents) are incompatible with in vitro assays using live cells, to determine its efficacy (Figure S6). Our solubility studies show that SR‐17018 (in either free base or mesylate salt form) has a higher saturating solubility in the solvent system used for in vivo assays (DMSO/Tween 80/water 4:16:80) compared to the corresponding in vitro solvent systems (DMSO/PBS 1:99). Furthermore, the mesylate salt form is relatively more soluble in DMSO/Tween 80/water (4:16:80) in comparison to the corresponding free base. Finally, our solubility data correlates with the work of others who reported that the solubility of SR‐17018 mesylate salt can be further increased with a higher proportion of DMSO used in their in vivo oral solvent system (DMSO/Tween 80/water 10:10:80, solubility reported as ~2.5 mg·ml^−1^ or ~5000 μM) (Grim et al., 2020). Our measurements determined maximal SR‐17018 solubility in the buffer used for live cell assays as approximately 10 μM. This limit may underlie the different maximal effect of the SR‐17018 mesylate salt as compared to the SR‐17018 free base in our in vitro measurements of agonist effect. When used as a mesylate salt, SR‐17018 displays a maximal effect similar to that of morphine and higher than that of oliceridine in both the Nb33 and arrestin‐3 recruitment assays, although for an accurate measurement of efficacy one would desire a greater window of solubility. The ability of SR‐17018 to cause significant respiratory depression when administered orally might, therefore, be expected from its relative intrinsic efficacy. This limited solubility and the potential of SR‐17018 to partition into the membrane may also underlie recent observations of non‐competitive pharmacology and atypical μ‐receptor phosphorylation patterns elicited by this compound (Fritzwanker et al., 2021; Stahl et al., 2021), despite the fact that it appears to bind to the orthosteric site (Zhuang et al., 2022) and therefore would be expected to act competitively.

Taking the above into account, the in vitro data presented in this study agrees with previous reports, with oliceridine displaying the lowest efficacy followed by morphine, oxycodone and tianeptine, while methadone displays the highest efficacy of the compounds tested in vivo. Nb33 is a measurement of μ‐receptor activation rather than G protein signalling, although our previous studies have shown that the relative efficacies of agonists detected using this approach predicted their relative efficacies in various measurements of μ‐receptor G protein signalling. These data were also analysed to determine whether biased agonism could be detected. No significant bias was detected for oliceridine, tianeptine or SR‐17018 (free base or mesylate salt) (Figures S8 and Table S5), between the activation of μ‐receptor (assessed using the Nb33 recruitment assay) and arrestin‐3 recruitment. Rather, the estimated transduction coefficients when normalised to the reference agonist DAMGO were not statistically different across these two assays.

In summary, our results show that all of the novel and reference opioid agonists, regardless of their putative pharmacodynamic differences, cause similar respiratory depression when used at high doses. Pre‐clinical studies designed to compare the relative safety of different opioids generally measure the ability of therapeutic doses (i.e., those that cause effective anti‐nociception) to induce side effects. While such studies may reveal a therapeutic window, they do not capture the potential of opioids to induce adverse side effects at doses beyond those producing effective anti‐nociception, which can be a useful additional measurement during their characterisation and provide a complementary and more comprehensive pre‐clinical assessment of opioid safety.

In conclusion, our data show that the opioid agonists oliceridine, tianeptine and SR‐17018, while providing effective anti‐nociception, significantly depress respiration in male mice with a similar effect at highest doses, albeit with different potencies and kinetics of such effect. These in vivo data are consistent with the in vitro μ‐receptor agonist activity of all the tested compounds. Accordingly, it is unlikely that these novel opioids are substantially differentiated from classical opioids with respect to their propensity to induce potentially life‐threatening respiratory depression at high doses. However, the differences in in vivo potency and kinetics of effect may be relevant to, and exploited for, their therapeutic application in different clinical settings.

## AUTHOR CONTRIBUTIONS

RH performed all animal behavioural experiments, analysed the data, prepared drafts of the figures. JS performed and analysed in vitro assays. SNM, MA and YH prepared the SR‐17018 mesylate salt and performed solubility studies. LL, ACK and JAJ provided reagents and assisted in study design. RH, JS, JRL and MC conceived the study and wrote the manuscript. All authors edited the manuscript.

## CONFLICT OF INTEREST STATEMENT

ACK is a co‐founder and shareholder of Kures, Inc. (a subsidiary of ATAI Life Sciences) and Gilgamesh Pharmaceuticals, Inc. Kures is currently developing mitragynine and tianeptine analogues as potential pharmaceuticals. ACK is named as an inventor on mitragynine‐ and tianeptine‐related patent applications owned by Columbia University and Kures. JAJ is a co‐founder of Kures and named as an inventor on mitragynine‐ and tianeptine‐related patents owned by Columbia University. No funding was provided by Kures for this work.

## DECLARATION OF TRANSPARENCY AND SCIENTIFIC RIGOUR

This Declaration acknowledges that this paper adheres to the principles for transparent reporting and scientific rigour of preclinical research as stated in the *BJP* guidelines for Design and Analysis and Animal Experimentation, and as recommended by funding agencies, publishers and other organisations engaged with supporting research.

## Supporting information


**Figure S1.** Raw minute volume (mL.min^−1^) values for morphine, tianeptine, oliceridine, SR‐17018, methadone and oxycodone dose response curves shown as percent of baseline MV in Figure 1 and Figure 2. n = 6 for all groups.
**Figure S2. (A)** Alternative regressions fit using AUC instead of maximum respiratory depression shown in Figure 3A. **(B)** Sigmoidal 3‐parameter fit of Figure 3A. n = 6 for each data point.
**Figure S3.** Raw minute volume (mL.min^−1^) **(A)** and raw hot plate latency (seconds) **(B)** values for morphine, tianeptine, oliceridine, SR‐17018, methadone and oxycodone dose response curves shown as percent of baseline MV in Figure 3 or as %MPE in Figure 4, respectively. n = 6 and n = 10 for plethysmography and hot plate, respectively.
**Figure S4. (A)** Raw minute volume (mL.min^−1^) and percent of baseline **(B)** values for mice administered SR‐17018 0.66 mg·kg^−1^ by oral or i.p administration and respective vehicles. **(C)** AUC analysis of **(B)** shows significant depression of respiration by oral but not i.p administered SR‐17018. **(D)** Raw hot plate latency (sec) and % maximum possible effect (%MPE) **(E)** for mice administered SR‐17018 0.66 mg·kg^−1^ by oral or i.p administration or vehicle. **(F)** AUC analysis of **(E)** shows significant antinociception by oral but not i.p. SR‐17018. Comparisons in **(C‐E)** made by Two‐way ANOVA with Bonferroni's comparison (**C**: F = 18.72; DFn = 1; DFd = 20) (**D**: F = 86.67; DFn = 2; DFd = 243), (**E**: F = 89.34; DFn = 2; DFd = 189). Comparison in **(F)** made by one‐way ANOVA with Bonferroni's comparison (**F**: F = 38.88; DFn = 2; DFd = 27). * indicates p < .05). n = 6 for **(A‐C)** and n = 10 **(D‐E)**. Statistical test details are provided in Supplementary Table 2.
**Figure S5. Induction of opioid respiratory depression in mice breathing 5% CO**_**2**_. Raw minute volume (mL.min^−1^) **(A),** percent of baseline MV **(B)** and AUC **(C)** values for calculated equi‐effective respiratory doses for morphine (3.44 mg.kg^−1^), tianeptine (12.74 mg.kg^−1^), oliceridine (0.745 mg.kg^−1^), oxycodone (0.71 mg.kg^−1^), methadone (2.5 mg.kg^−1^) and SR‐17018 (0.66 mg.kg^−1^). Comparisons in (**B)** were made by one‐way ANOVA using Tukey's comparison. * indicates significance to vehicle control, p < .05. n = 6 for all groups. Statistical test details are provided in Supplementary Table 2.
**Figure S6. Effect of different buffers on live cell BRET measurements.** Cells were treated with 10% DMSO, 4% DMSO‐16% Tween (in vivo vehicle), 0.1% DMSO (in vitro vehicle) or 10 μM DAMGO. The effect of each addition on the raw intensity counts for bioluminescence (475 nm) and fluorescence (535 nm) emission wavelengths was measured over time. Data show mean ± SEM of n = 6 experiments performed in duplicate.
**Figure S7. Standard calibration curves for SR‐17018 in LCMS analysis** (See Materials and Methods). SR‐17018 concentration range 0.61–620.86 μM; area under the curve (AUC) axis has arbitrary units.
**Figure S8. Biased agonism quantification.** Biased agonism was quantified as previously described (Kenakin et al., 2012) using predefined equations in GraphPad Prism 9.5.0 to determine transduction coefficients (Log[τ/K_A_]) for each agonist at each pathway. **A‐G** Normalised transduction coefficients (ΔLog[τ/K_A_]) for each agonist in each assay were determined by subtracting the transduction coefficient for DAMGO on the corresponding plate from the transduction coefficient of each agonist. ΔLog[τ/K_A_] values for each agonist at each assay point were determined from 5 separate experimental repeats. These values were used to determine the mean ΔLog[τ/K_A_] values for each agonist at each pathway. The mean ΔLog[τ/K_A_] values for a particular agonist were compared between Nb33 and arrestin‐3 recruitment using an unpaired two‐tailed Students t‐test, * indicates significant difference p < 0.05. A significant difference in ΔLog[τ/K_A_] values for a particular agonist for arrestin‐3 recruitment relative to Nb33 recruitment is indicative of bias. **H.** The LogBias factor (ΔΔLog[τ/K_A_])) was determined by calculating the difference between the ΔLog[τ/K_A_] values for each agonist between the two signalling assays.

**Table S1.** Maximum induced depression of minute volume (MV), tidal volume (TV) and respiratory frequency (F) expressed as a percentage of baseline MV, by doses of morphine, tianeptine, oliceridine (all dosed i.p.), and methadone, oxycodone and SR‐17018 (all dosed p.o.).
**Table S2.** Statistical analysis.
**Table S3.** pEC_50_ and E_max_ values for Nb33 and arrestin‐3 recruitment assays. E_max_ values are calculated as a percentage of the maximal effect (E_max_) of DAMGO. All values represent the mean ± SEM of 5 experiments. * indicates p < 0.05 compared to DAMGO, one‐way ANOVA with Tukey's comparison for each column.
**Table S4.** Raw AUC data for each SR‐17018 and SR‐17018 mesylate salt in each solvent system tested. Where possible, the mean and standard deviation (SD) across the set of three samples was calculated. The slope (1838.76) and y‐intercept (5371.88) parameters for the SR‐17018 calibration curve were used to calculate mean saturation concentration, taking into account the 1 in 10 dilution of samples after filtration. For some solvent conditions, sample solubility was too low to allow consistent detection to take place within the detection limits of the system (ND).
**Table S5. Biased agonism quantification.** Biased agonism was quantified as previously described (Kenakin et al., 2012) using predefined equations in GraphPad Prism 9.5.0 to determine transduction coefficients (Log[τ/K_A_]) for each agonist at each pathway. Normalised transduction coefficients (ΔLog[τ/K_A_]) for each agonist in each assay were determined by subtracting the transduction coefficient for DAMGO on the corresponding plate from the transduction coefficient of each agonist. ΔLog[τ/K_A_] values for each agonist at each assay point were determined from 5 separate experimental repeats. These values were used to determine the mean ΔLog[τ/K_A_] values for each agonist at each pathway. The mean ΔLog[τ/K_A_] values for a particular agonist were compared between Nb33 and arrestin recruitment as shown in Supplementary Figure 8. A significant difference in ΔLog[τ/K_A_] values for a particular agonist for arrestin recruitment relative to Nb33 recruitment is indicative of bias (Supp Figure 8). The LogBias factor (ΔΔLog[τ/K_A_])) was determined by calculating the difference between the ΔLog[τ/K_A_] values for each agonist between the two signalling assays.

## Data Availability

The data that support the findings of this study are available from the corresponding authors upon reasonable request.
