# Supplementary material for: Assessment of the potential of novel and classical opioids to induce respiratory depression in mice
Source: Br J Pharmacol. 2023 Aug 22;180(24):3160–74. doi: 10.1111/bph.16199 (PMC10952895; doi:10.1111/bph.16199)
Supplement: Supplementary file 1 — Figure S1. Raw minute volume (mL.min−1) values for morphine, tianeptine, oliceridine, SR‐17018, methadone and oxycodone dose response curves shown as percent of baseline MV in Figure 1 and Figure 2. n = 6 for all groups. Figure S2. (A) Alternative regressions fit using AUC instead of maximum respiratory depression shown in Figure 3A. (B) Sigmoidal 3‐parameter fit of Figure 3A. n = 6 for each data point. Figure S3. Raw minute volume (mL.min−1) (A) and raw hot plate latency (seconds) (B) values for morphine, tianeptine, oliceridine, SR‐17018, methadone and oxycodone dose response curves shown as percent of baseline MV in Figure 3 or as %MPE in Figure 4, respectively. n = 6 and n = 10 for plethysmography and hot plate, respectively. Figure S4. (A) Raw minute volume (mL.min−1) and percent of baseline (B) values for mice administered SR‐17018 0.66 mg·kg−1 by oral or i.p administration and respective vehicles. (C) AUC analysis of (B) shows significant depression of respiration by oral but not i.p administered SR‐17018. (D) Raw hot plate latency (sec) and % maximum possible effect (%MPE) (E) for mice administered SR‐17018 0.66 mg·kg−1 by oral or i.p administration or vehicle. (F) AUC analysis of (E) shows significant antinociception by oral but not i.p. SR‐17018. Comparisons in (C‐E) made by Two‐way ANOVA with Bonferroni's comparison (C: F = 18.72; DFn = 1; DFd = 20) (D: F = 86.67; DFn = 2; DFd = 243), (E: F = 89.34; DFn = 2; DFd = 189). Comparison in (F) made by one‐way ANOVA with Bonferroni's comparison (F: F = 38.88; DFn = 2; DFd = 27). * indicates p < .05). n = 6 for (A‐C) and n = 10 (D‐E). Statistical test details are provided in Supplementary Table 2. Figure S5. Induction of opioid respiratory depression in mice breathing 5% CO 2 . Raw minute volume (mL.min−1) (A), percent of baseline MV (B) and AUC (C) values for calculated equi‐effective respiratory doses for morphine (3.44 mg.kg−1), tianeptine (12.74 mg.kg−1), oliceridine (0.745 mg.kg−1), oxycodone (0.71 mg.kg−1), [file BPH-180-3160-s001.pdf]

Supp Fig 1

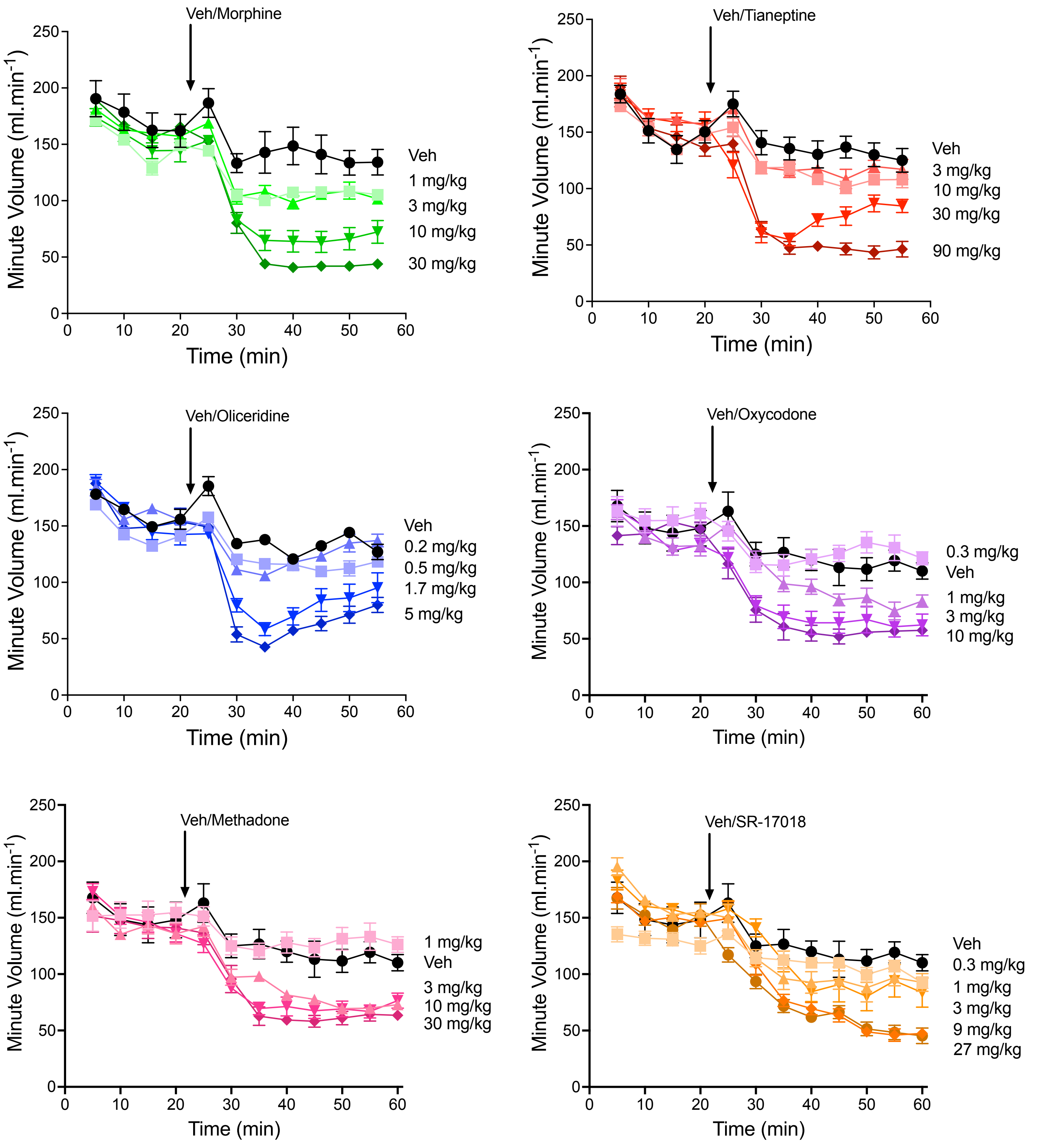

Supplementary Figure 1. Raw minute volume (mL.min^{-1}) values for morphine, tianeptine, oliceridine, SR-17018, methadone and oxycodone dose response curves shown as percent of baseline MV in Figure 1 and Figure 2. N=6 for all groups.

Supp Fig 2

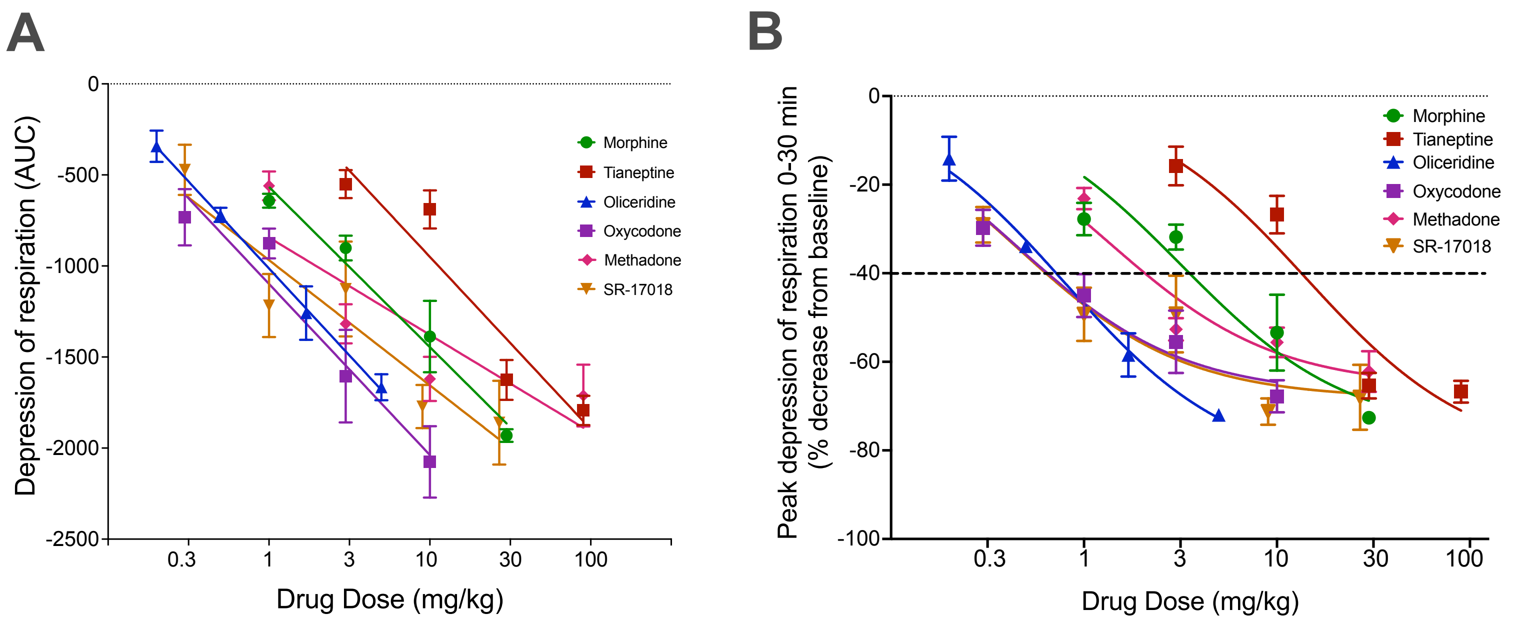

Supplementary Figure 2. **(A)** Alternative regressions fit using AUC instead of maximum respiratory depression shown in Fig 3A. **(B)** Sigmoidal 3-parameter fit of Fig 3A. N=6 for each data point.

Supp Fig 3

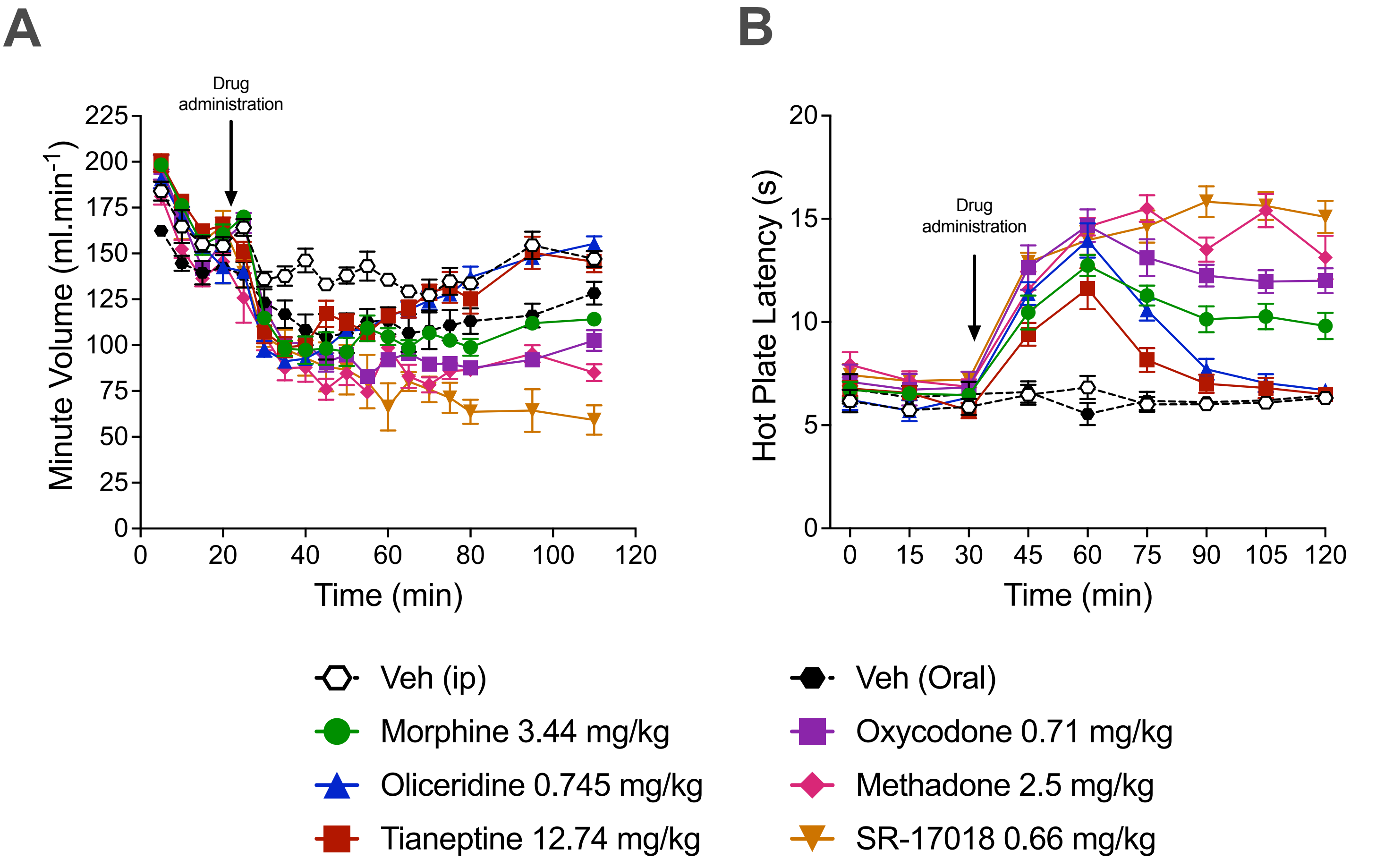

Supplementary Figure 3. Raw minute volume (mL.min^{-1}) **(A)** and raw hot plate latency (seconds) **(B)** values for morphine, tianeptine, oliceridine, SR-17018, methadone and oxycodone dose response curves shown as percent of baseline MV in Figure 3 or as %MPE in Figure 4, respectively. N=6 and N=10 for plethysmography and hot plate, respectively.

Supp Fig 4

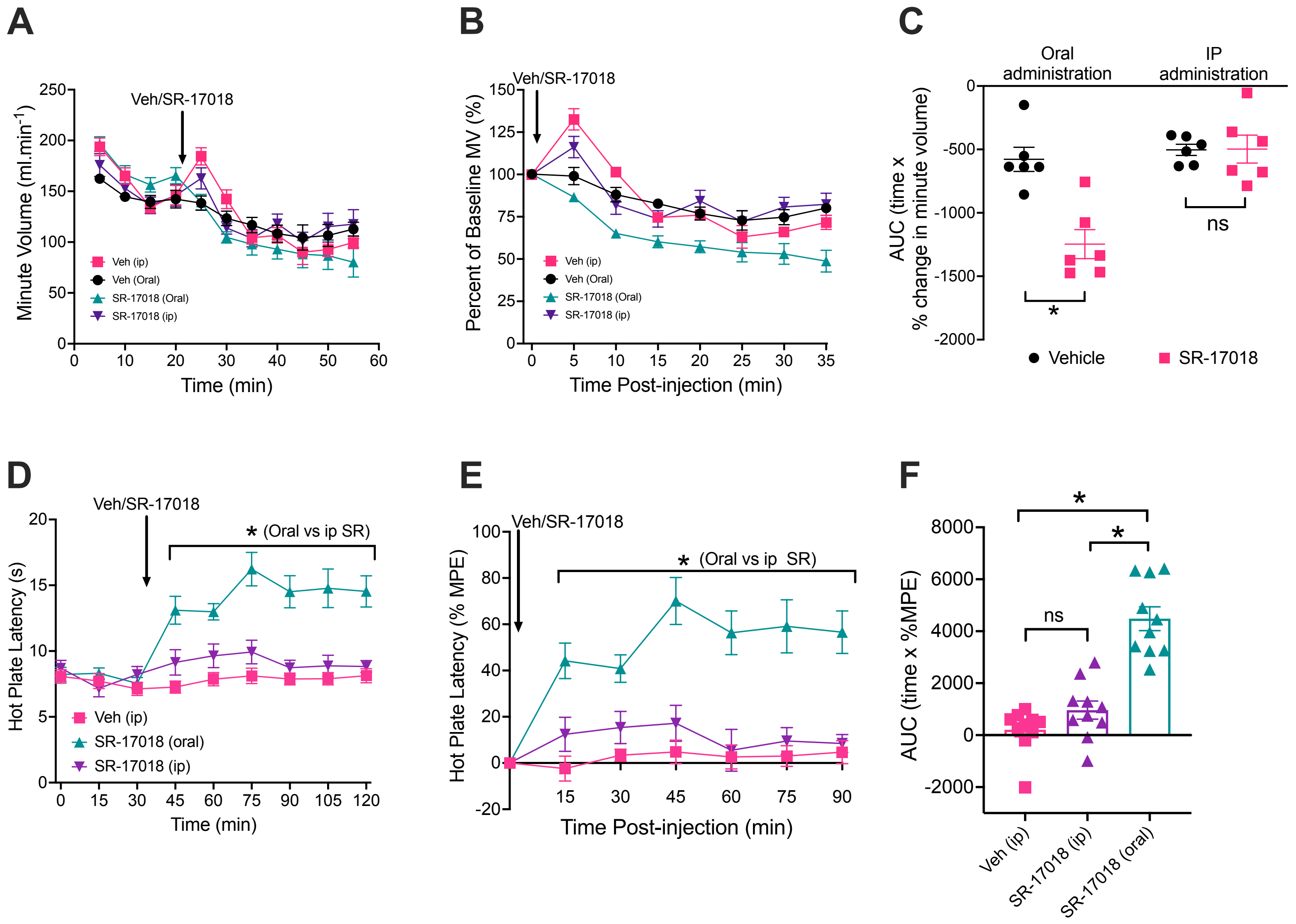

Supplementary Figure 4. (A) Raw minute volume (mL.min^{-1}) and percent of baseline **(B)** values for mice administered SR-17018 0.66 mg/kg by oral or i.p administration and respective vehicles. **(C)** AUC analysis of (B) shows significant depression of respiration by oral but not i.p administered SR-17018. **(D)** Raw hot plat latency (sec) and % maximum possible effect (%MPE) **(E)** for mice administered SR-17018 0.66 mg/kg by oral or i.p administration or vehicle. **(F)** AUC analysis of **(E)** shows significant antinociception by oral but not i.p. SR-17018 Comparisons in **(C-E)** made by Two-way ANOVA with Tukey's comparison. Comparison in **(F)** made by One-way ANOVA with Tukey's comparison). * indicates p<0.05). N=6 for **(A-C)** and N=10 **(D-E)**. Statistical test details are provided in Supplementary Table 2.

Supp Fig 5

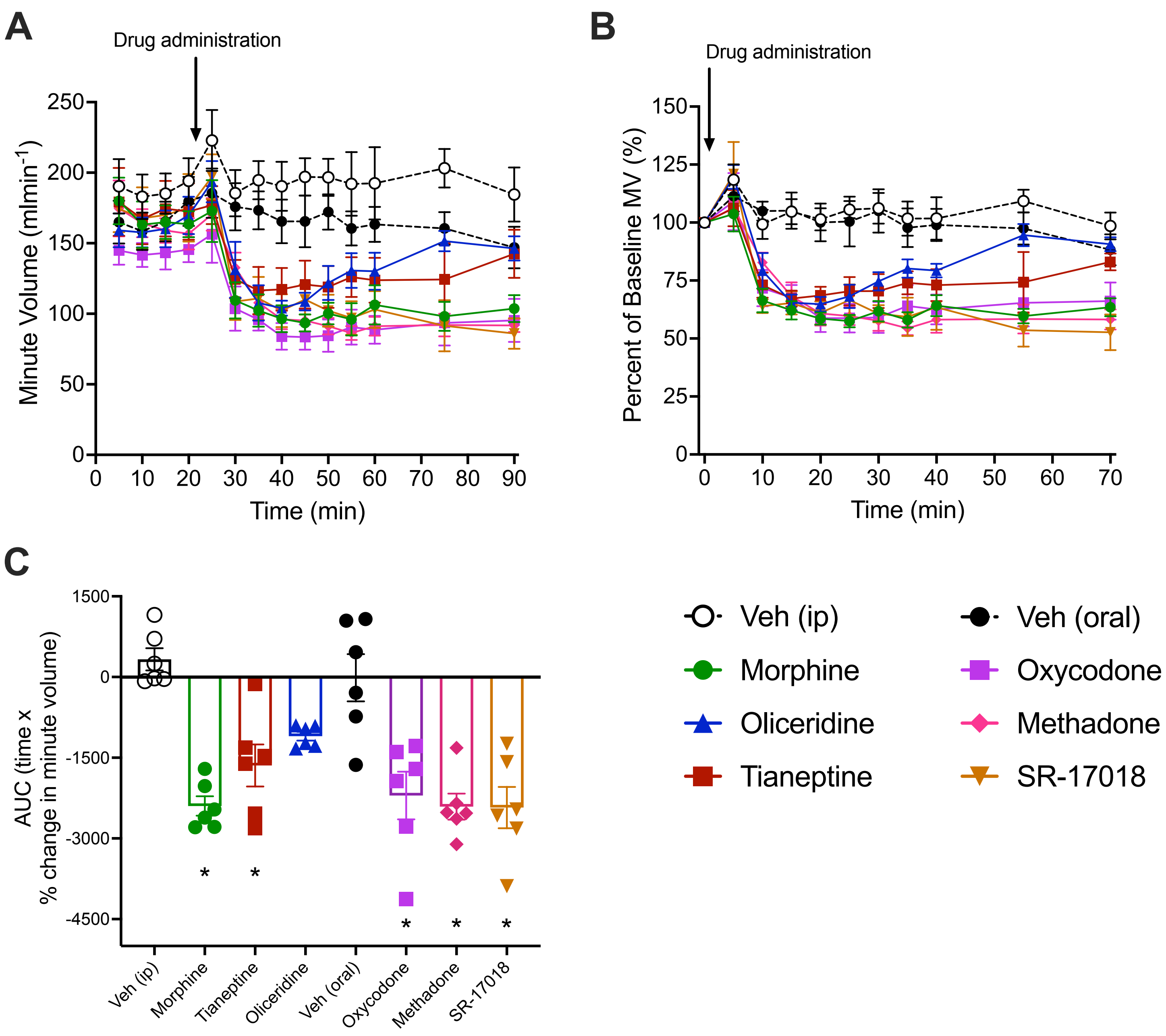

Supplementary Figure 5. Induction of opioid respiratory depression in mice breathing 5% CO_2. Raw minute volume (ml.min^{-1}) **(A),** percent of baseline MV **(B)** and AUC **(C)** values for calculated equi-effective respiratory doses for morphine (3.44 mg.kg^{-1}), tianeptine (12.74 mg.kg^{-1}), oliceridine (0.745 mg.kg^{-1}), oxycodone (0.71 mg.kg^{-1}), methadone (2.5 mg.kg^{-1}) and SR-17018 (0.66 mg.kg^{-1}). Comparisons in **B** were made by One-way ANOVA using Tukey's comparison. * indicates significance to vehicle control, p<0.05. N=6 for all groups. Statistical test details are provided in Supplementary Table 2.

Supp Fig 6

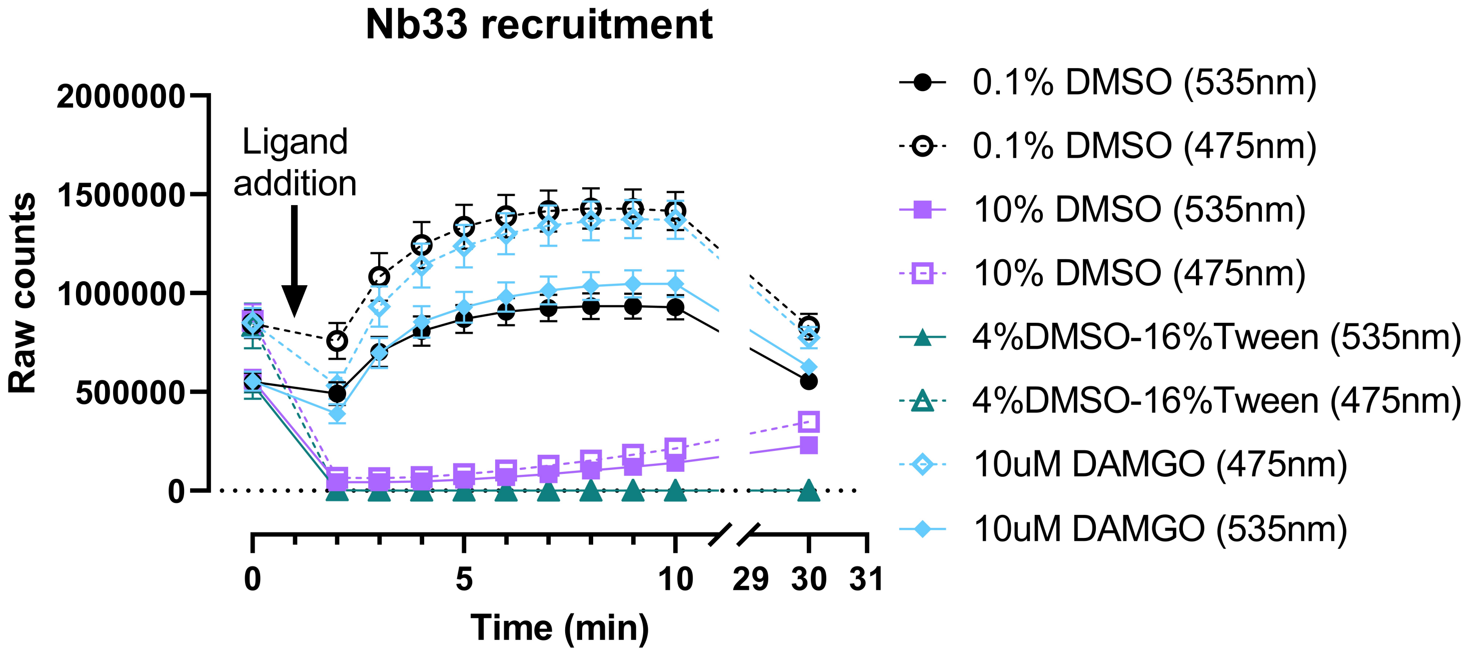

Supplementary Figure 6. Effect of different buffers on live cell BRET measurements. Cells were treated with 10% DMSO, 4% DMSO-16% Tween (in vivo vehicle), 0.1% DMSO (in vitro vehicle) or 10 µM DAMGO. The effect of each addition on the raw intensity counts for both bioluminescence (475 nm) and fluorescence (535 nm) emission wavelengths was measured over time. Data show mean ± SEM of N=6 experiments performed in duplicate.

Supp Fig 7

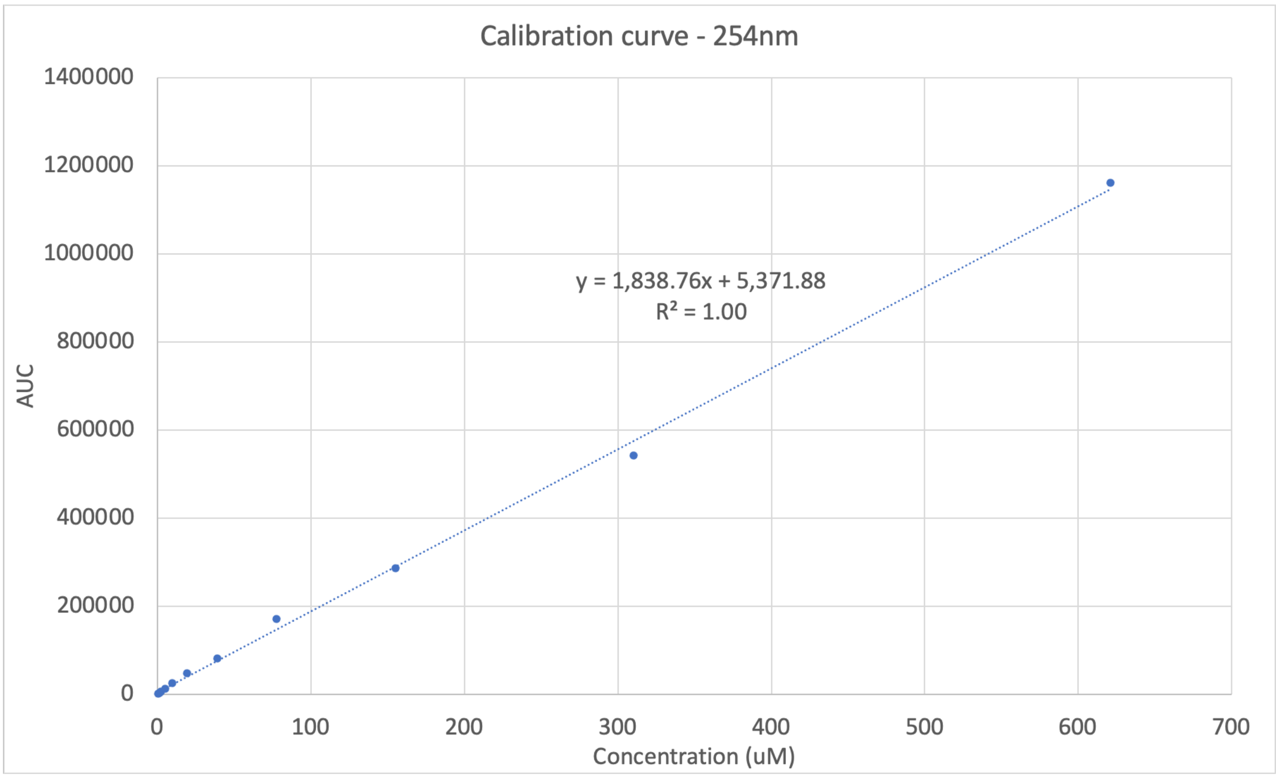

Supplementary Figure 7. Standard calibration curves for SR-17018 in HPLC LCMS analysis (See Materials and Methods). SR-17018 concentration range 0.61 – 620.86 µM; area under the curve (AUC) axis has arbitrary units.

Supp Fig 8

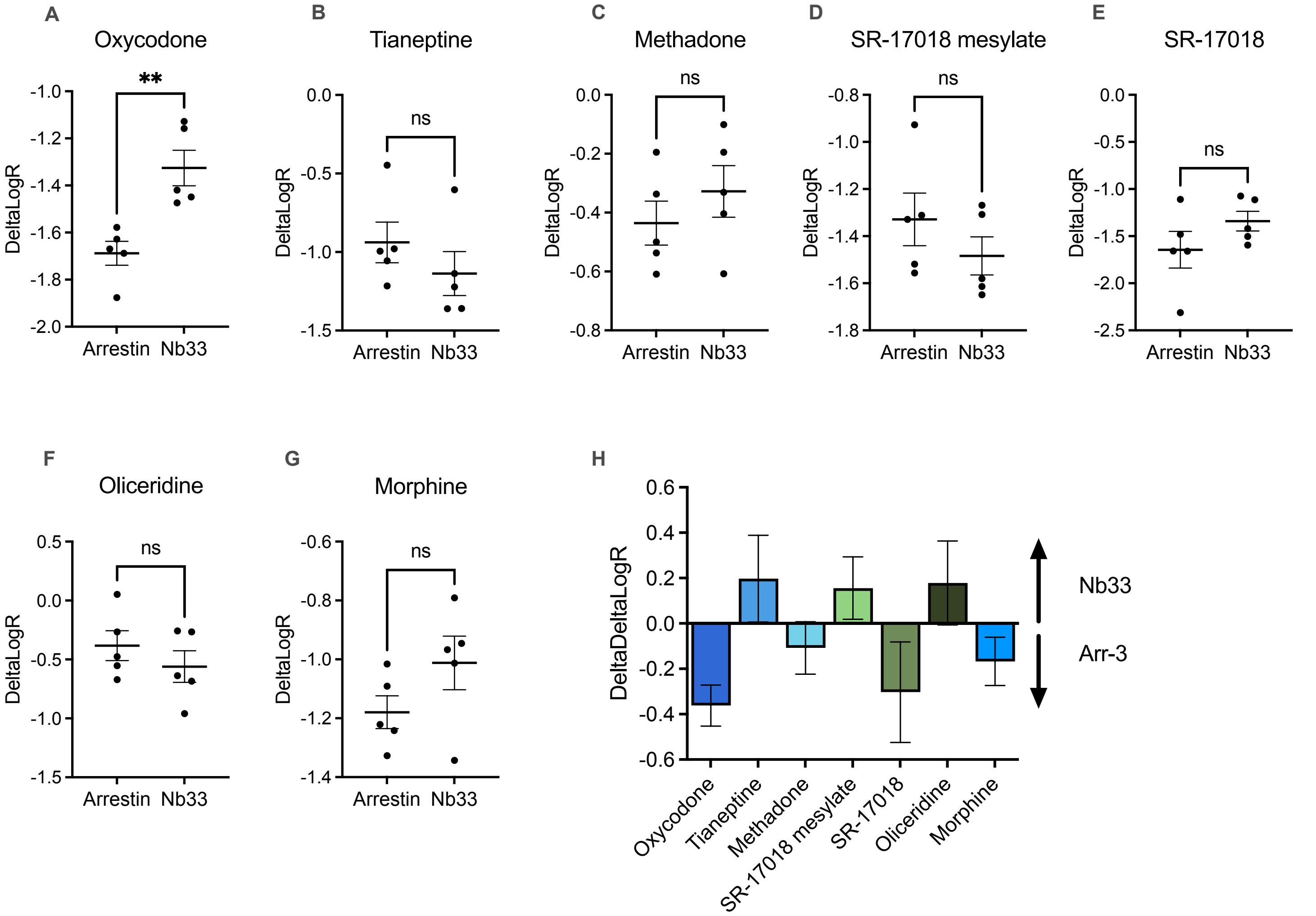

Supplementary Figure 8. Biased agonism quantification. Biased agonism was quantified as previously described (Kenakin et al., 2012) using predefined equations in GraphPad Prism 9.5.0 to determine transduction coefficients ($Log[\tau/K_A]$) for each agonist at each pathway. **A-G** Normalised transduction coefficients ($\Delta Log[\tau/K_A]$) for each agonist in each assay were determined by subtracting the transduction coefficient for DAMGO on the corresponding plate from the transduction coefficient of each agonist. $\Delta Log[\tau/K_A]$ values for each agonist at each assay point were determined from 5 separate experimental repeats. These values were used to determine the mean $\Delta Log[\tau/K_A]$ values for each agonist at each pathway. The mean $\Delta Log[\tau/K_A]$ values for a particular agonist were compared between Nb33 and arrestin-3 recruitment using an unpaired two-tailed Students t-test, * indicates significant difference $p < 0.05$. A significant difference in $\Delta Log[\tau/K_A]$ values for a particular agonist for arrestin-3 recruitment relative to Nb33 recruitment is indicative of bias. **H.** The LogBias factor ($\Delta\Delta Log[\tau/K_A]$)) was determined by calculating the difference between the $\Delta Log[\tau/K_A]$ values for each agonist between the two signalling assays.
