# Supplementary material for: Assessment of the potential of novel and classical opioids to induce respiratory depression in mice
Source: Br J Pharmacol. 2023 Aug 22;180(24):3160–74. doi: 10.1111/bph.16199 (PMC10952895; doi:10.1111/bph.16199)
Supplement: Supplementary file 2 — Table S1. Maximum induced depression of minute volume (MV), tidal volume (TV) and respiratory frequency (F) expressed as a percentage of baseline MV, by doses of morphine, tianeptine, oliceridine (all dosed i.p.), and methadone, oxycodone and SR‐17018 (all dosed p.o.). Table S2. Statistical analysis. Table S3. pEC50 and Emax values for Nb33 and arrestin‐3 recruitment assays. Emax values are calculated as a percentage of the maximal effect (Emax) of DAMGO. All values represent the mean ± SEM of 5 experiments. * indicates p < 0.05 compared to DAMGO, one‐way ANOVA with Tukey's comparison for each column. Table S4. Raw AUC data for each SR‐17018 and SR‐17018 mesylate salt in each solvent system tested. Where possible, the mean and standard deviation (SD) across the set of three samples was calculated. The slope (1838.76) and y‐intercept (5371.88) parameters for the SR‐17018 calibration curve were used to calculate mean saturation concentration, taking into account the 1 in 10 dilution of samples after filtration. For some solvent conditions, sample solubility was too low to allow consistent detection to take place within the detection limits of the system (ND). Table S5. Biased agonism quantification. Biased agonism was quantified as previously described (Kenakin et al., 2012) using predefined equations in GraphPad Prism 9.5.0 to determine transduction coefficients (Log[τ/KA]) for each agonist at each pathway. Normalised transduction coefficients (ΔLog[τ/KA]) for each agonist in each assay were determined by subtracting the transduction coefficient for DAMGO on the corresponding plate from the transduction coefficient of each agonist. ΔLog[τ/KA] values for each agonist at each assay point were determined from 5 separate experimental repeats. These values were used to determine the mean ΔLog[τ/KA] values for each agonist at each pathway. The mean ΔLog[τ/KA] values for a particular agonist were compared between Nb33 and arrestin recruitment as shown in Supplementary Figur [file BPH-180-3160-s002.docx]

**Supplementary Table 1.** Maximum induced depression of minute volume (MV), tidal volume (TV) and respiratory frequency (F) expressed as a percentage of baseline MV, by doses of morphine, tianeptine, oliceridine (all dosed i.p.), and methadone, oxycodone and SR-17018 (all dosed p.o.).

| **Drug and dose** | **Max. effect on MV (% baseline) ± s.e.m** | **Max. effect on TV (% baseline) ± s.e.m** | **Max. effect on F (% baseline) ± s.e.m** | **N number** |
| --- | --- | --- | --- | --- |
| Morphine 1 mg.kg^-1^ | -27.8 ± 3.6 | -7.8 ± 1.5 | -32.6 ± 1.4 | 6 |
| Morphine 3 mg.kg^-1^ | -31.8 ± 2.8 | -10.5 ± 1.9 | -51.5 ± 9.9 | 6 |
| Morphine 10 mg.kg^-1^ | -53.4 ± 8.6 | -5.6 ± 2.8 | -61.3 ± 6.2 | 6 |
| Morphine 30 mg.kg^-1^ | -72.6 ± 0.7 | -14.2 ± 5.0 | -72.1 ± 2.2 | 6 |
|  |  |  |  |  |
| Tianeptine 3 mg.kg^-1^ | -15.8 ± 4.4 | -10.9 ± 2.6 | -30.6 ± 3.4 | 6 |
| Tianeptine 10 mg.kg^-1^ | -26.8 ± 4.3 | -3.8 ± 3.4 | -35.7 ± 5.5 | 6 |
| Tianeptine 30 mg.kg^-1^ | -65.4 ± 2.9 | -16.8 ± 5.4 | -61.6 ± 3.1 | 6 |
| Tianeptine 90 mg.kg^-1^ | -66.7 ± 2.5 | -18.0 ± 5.4 | -64.3 ± 8.4 | 6 |
|  |  |  |  |  |
| Oliceridine 0.2 mg.kg^-1^ | -14.1 ± 4.9 | -5.1 ± 3.4 | -30.2 ± 3.8 | 6 |
| Oliceridine 0.5 mg.kg^-1^ | -33.9 ± 1.0 | -4.0 ± 3.9 | -35.1 ± 3.1 | 6 |
| Oliceridine 1.7 mg.kg^-1^ | -58.5 ± 4.9 | -16.7 ± 2.8 | -55.5 ± 4.7 | 6 |
| Oliceridine 5 mg.kg^-1^ | -72.0 ± 1.3 | -13.6 ± 5.5 | -67.9 ± 2.2 | 6 |
|  |  |  |  |  |
| Methadone 1 mg.kg^-1^ | -23.2 ± 2.4 | -10.8 ± 6.5 | -25.6 ± 4.7 | 6 |
| Methadone 3 mg.kg^-1^ | -52.7 ± 2.5 | -14.5 ± 3.8 | -47.9 ± 3.8 | 6 |
| Methadone 10 mg.kg^-1^ | -55.6 ± 3.3 | -20.1 ± 6.1 | -51.4 ± 4.2 | 6 |
| Methadone 30 mg.kg^-1^ | -62.2 ± 4.6 | -16.9 ± 6.0 | -58.6 ± 5.8 | 6 |
|  |  |  |  |  |
| Oxycodone 0.3 mg.kg^-1^ | -29.7 ± 4.1 | -4.9 ± 2.7 | -31.1 ± 2.7 | 6 |
| Oxycodone 1 mg.kg^-1^ | -45.0 ± 4.9 | -4.1 ± 4.3 | -45.5 ± 4.8 | 6 |
| Oxycodone 3 mg.kg^-1^ | -55.5 ± 7.0 | -22.2 ± 8.2 | -54.0 ± 4.9 | 6 |
| Oxycodone 10 mg.kg^-1^ | -67.8 ± 3.6 | -22.4 ± 9.5 | -64.3 ± 5.6 | 6 |
|  |  |  |  |  |
| SR-17018 0.3 mg.kg^-1^ | -29.1 ± 4.0 | -11.8 ± 2.2 | -32.0 ± 3.9 | 6 |
| SR-17018 1 mg.kg^-1^ | -49.3 ± 6.0 | -23.5 ± 5.9 | -32.3 ± 8.3 | 6 |
| SR-17018 3 mg.kg^-1^ | -49.2 ± 8.7 | -5.4 ± 5.5 | -53.6 ± 7.7 | 6 |
| SR-17018 9 mg.kg^-1^ | -71.3 ± 3.0 | -21.7 ± 7.2 | -71.3 ± 3.0 | 6 |
| SR-17018 27 mg.kg^-1^ | -68.0 ± 7.3 | -13.6 ± 3.4 | -69.0 ± 4.7 | 6 |

**Supplementary Table 2 – Statistical analysis.**

| **Figure** | **Section** | **Test** | **Details (F(DFn,DFd)** |
| --- | --- | --- | --- |
| Figure 1 | **B** | One-way ANOVA with Tukey’s multiple comparison | ANOVA result (between columns): F (4, 25) = 32.92, p<0.0001 |
|  | **D** |  | ANOVA result (between columns): F (4, 25) = 42.33, p<0.0001 |
|  | **F** |  | ANOVA result (between columns): F (4, 25) = 39.91, p<0.0001 |
| Figure 2 | **B** | One-way ANOVA with Tukey’s multiple comparison | ANOVA result (between columns): F (4, 25) = 14.44, p<0.0001 |
|  | **D** |  | ANOVA result (between columns): F (4, 25) = 21.53, p<0.0001 |
|  | **F** |  | ANOVA result (between columns): F (4, 25) = 10.62, p<0.0001 |
| Figure 3 | **B** | Two-way ANOVA with Tukey’s multiple comparisons (Factors = time and drug) | Interaction: F (98, 560) = 8.473, p<0.0001  Row (time): F (7.467, 298.7) = 119.8, p<0.0001  Column (drug): F (7, 40) = 16.67, p<0.0001 |
|  | **C** | One-way ANOVA with Tukey’s multiple comparison | ANOVA result (between columns): F (5, 30) = 1.051, p=0.4064 |
|  | **D** |  | ANOVA result (between columns): F (7, 40) = 18.76, p<0.0001 |
| Figure 4 | **A** | Two-way ANOVA with Tukey’s multiple comparisons (Factors = time and dose) | Interaction: F (42, 432) = 10.74, p<0.0001  Row (time): F (4.292, 309) = 80.31, p<0.0001  Column (dose): F (7, 72) = 57.67, p<0.0001 |
|  | **B** | One-way ANOVA with Tukey’s multiple comparison | ANOVA result (between columns): F (5, 54) = 2.492, p=0.0421 |
|  | **C** |  | ANOVA result (between columns): F (7, 72) = 60.11, p<0.0001 |

| **Figure** | **Section** | **Test** | **Details (F(DFn,DFd)** |
| --- | --- | --- | --- |
| Supp. Figure 4 | **C** | One-way ANOVA with Tukey’s multiple comparison | ANOVA result (between columns): F (7, 40) = 12.04, p<0.0001 |
| Supp. Figure 5 | **C** | Two-way ANOVA with Tukey’s multiple comparisons (Factors = drug and administration route) | Interaction: F (1, 20) = 12.53, p=0.0021  Row (admin route): F (1, 20) = 18.72, p=0.0003  Column (drug): F (1, 20) = 12.10, p=0.0024 |
|  | **D** | Two-way ANOVA with Tukey’s multiple comparisons (Factors = time and administration route) | Interaction: F (16, 243) = 5.16, p<0.0001  Row (time): F (8, 243) = 9.41, p<0.0001  Column (admin route): F (2, 243) = 82.67, p<0.0001 |
|  | **E** | Two-way ANOVA with Tukey’s multiple comparisons (Factors = time and administration route) | Interaction: F (12, 560) = 3.24, p=0.0003  Row (time): F (6, 298.7) = 6.15, p<0.0001  Column (admin route): F (2, 40) = 89.34, p<0.0001 |
|  | **F** | One-way ANOVA with Tukey’s multiple comparison | ANOVA result (between columns): F (2, 27) = 38.88, p<0.0001 |
| Supp Table 3 | **Nb33 pEC_50_** | One-way ANOVA with Tukey’s multiple comparison | ANOVA result (between columns): F (7, 32) = 23.16, p<0.0001 |
|  | **Nb33 E_max_** | One-way ANOVA with Tukey’s multiple comparison | ANOVA result (between columns): F (7, 32) = 83.65, p<0.0001 |
|  | **Arr3 pEC_50_** | One-way ANOVA with Tukey’s multiple comparison | ANOVA result (between columns): F (7, 32) = 145.6, p<0.0001 |
|  | **Arr3 E_max_** | One-way ANOVA with Tukey’s multiple comparison | ANOVA result (between columns): F (7, 32) = 52.73, p<0.0001 |

**Supplementary Table 3.** pEC_50_ and E_max_ values for Nb33 and arrestin-3 recruitment assays. E_max_ values are calculated as a percentage of the maximal effect (E_max_) of DAMGO. All values represent the mean ± SEM of 5 experiments. * indicates p<0.05 compared to DAMGO, one-way ANOVA with Tukey’s comparison for each column.

| **Drug** | **Nb33 recruitment pEC_50_ ± SEM [EC_50_, nM]** | **Nb33 recruitment E_max_ ± SEM (%)** | **Arr3 recruitment pEC_50_ ± SEM [EC_50_, nM]** | **Arr3 recruitment E_max_ ± SEM (%)** |
| --- | --- | --- | --- | --- |
| DAMGO | 6.8 ± 0.04 [150] | 99 ± 2 | 7.6 ± 0.03 [28] | 100 ± 1 |
| Morphine | 6.0 ± 0.07 [894]* | 67 ± 3 * | 6.5 ± 0.04 [317]* | 77 ± 1* |
| Tianeptine | 5.8 ± 0.07 [1415]* | 70 ± 3 * | 6.6 ± 0.03 [239]* | 96 ± 1 |
| Oliceridine | 6.8 ± 0.1 [149] | 33 ± 2 * | 7.4 ± 0.06 [37] | 52 ± 1* |
| Oxycodone | 5.8 ± 0.05 [1532]* | 68 ± 2 * | 6.1 ± 0.02 [864]* | 78 ± 1* |
| Methadone | 6.5 ± 0.05 [327] | 91 ± 2 | 7.1 ± 0.03 [87]* | 98 ± 1 |
| SR-17018 | 6.3 ± 0.2 [455]* | 18 ± 1 * | 5.9 ± 0.1 [1136]* | 85 ± 5* |
| SR mesylate | 5.6 ± 0.1 [2613]* | 59 ± 6* | 6.2 ± 0.06 [676]* | 96 ± 3 |

**Supplementary Table 4.** Raw AUC data for each SR-17018 and SR-17018 mesylate salt in each solvent system tested. Where possible, the mean and standard deviation (SD) across the set of three samples was calculated. The slope (1838.76) and y-intercept (5371.88) parameters for the SR-17018 calibration curve were used to calculate mean saturation concentration, taking into account the 1 in 10 dilution of samples after filtration. For some solvent conditions, sample solubility was too low to allow consistent detection to take place within the detection limits of the system (ND).

|  | **SR-17018 free base** | **SR-17018 mesylate salt** |
| --- | --- | --- |
|  |  |  |
| **DMSO/PBS (1:99)** |  |  |
| Sample 1 | 3347 | ND |
| Sample 2 | ND | 3650 |
| Sample 3 | ND | 1916 |
| Mean AUC | - | 2783 |
| SD | - | 1226 |
| Mean ± SD saturation concentration after accounting for dilution (μM) | 15.3 | 12.3 ± 3.7 |
|  |  |  |
| **DMSO/PBS (10:90)** |  |  |
| Sample 1 | 7021 | 20418 |
| Sample 2 | 12159 | 15686 |
| Sample 3 | ND | 70201 |
| Mean AUC | 9590 | 35435 |
| SD | 3633 | 30201 |
| Mean ± SD saturation concentration after accounting for dilution (μM) | 49.2 ± 16.8 | 190.0 ± 161.3 |
|  |  |  |
| **DMSO/Tween 80/MilliQ water (4:16:80)** |  |  |
| Sample 1 | 76429 | 775560 |
| Sample 2 | 70543 | 491290 |
| Sample 3 | 84897 | 596140 |
| Mean AUC | 77290 | 620997 |
| SD | 7216 | 143756 |
| Mean ± SD saturation concentration after accounting for dilution (μM) | 417.4 ± 36.3 | 3374.3 ± 778.9 |

**Supplementary Table 5. Biased agonism quantification.** Biased agonism was quantified as previously described (Kenakin et al., 2012) using predefined equations in GraphPad Prism 9.5.0 to determine transduction coefficients (Log[τ/K_A_]) for each agonist at each pathway. Normalised transduction coefficients (ΔLog[τ/K_A_]) for each agonist in each assay were determined by subtracting the transduction coefficient for DAMGO on the corresponding plate from the transduction coefficient of each agonist. ΔLog[τ/K_A_] values for each agonist at each assay point were determined from 5 separate experimental repeats. These values were used to determine the mean ΔLog[τ/K_A_] values for each agonist at each pathway. The mean ΔLog[τ/K_A_] values for a particular agonist were compared between Nb33 and arrestin recruitment as shown in Supplementary Fig 8. A significant difference in ΔLog[τ/K_A_] values for a particular agonist for arrestin recruitment relative to Nb33 recruitment is indicative of bias (Supp Fig 8). The LogBias factor (ΔΔLog[τ/K_A_])) was determined by calculating the difference between the ΔLog[τ/K_A_] values for each agonist between the two signalling assays.

| **βARR+GRK2** | | **Oxycodone** | **DAMGO** | **Tianeptine** | **Methadone** | **SR-17018**  **mesylate** | **SR-17018** | **Oliceridine** | **Morphine** |
| --- | --- | --- | --- | --- | --- | --- | --- | --- | --- |
| Log[τ/K_A_] Rep 1 | pl1 | 5.973 | 7.551 | 6.556 | 7.051 |  |  |  |  |
|  | pl2 |  | 7.406 |  |  | 6.479 | 6.296 | 7.458 | 6.185 |
| ΔLog[τ/K_A_] Rep 1 | | -1.578 | 0 | -0.995 | -0.5 | -0.927 | -1.11 | 0.052 | -1.221 |
|  |  |  |  |  |  |  |  |  |  |
| Log[τ/K_A_] Rep 2 | pl1 | 5.964 | 7.84 |  |  | 6.284 | 5.529 |  |  |
|  | pl2 |  | 7.618 | 6.562 | 7.009 |  |  | 6.947 | 6.602 |
| ΔLog[τ/K_A_] Rep 2 | | -1.876 | 0 | -1.056 | -0.609 | -1.556 | -2.311 | -0.671 | -1.016 |
|  |  |  |  |  |  |  |  |  |  |
| Log[τ/K_A_] Rep 3 | pl1 |  | 7.255 | 6.808 | 7.06 | 5.944 | 5.774 |  |  |
|  | pl2 | 5.974 | 7.662 |  |  |  |  | 7.109 | 6.335 |
| ΔLog[τ/K_A_] Rep 3 | | -1.688 | 0 | -0.447 | -0.195 | -1.311 | -1.481 | -0.553 | -1.327 |
|  |  |  |  |  |  |  |  |  |  |
| Log[τ/K_A_] Rep 4 | pl1 |  | 7.485 |  | 7.148 | 6.156 | 5.827 | 7.22 | 6.243 |
|  | pl2 | 5.855 | 7.525 | 6.546 |  |  |  |  |  |
| ΔLog[τ/K_A_] Rep 4 | | -1.67 | 0 | -0.979 | -0.337 | -1.329 | -1.658 | -0.265 | -1.242 |
|  |  |  |  |  |  |  |  |  |  |
| Log[τ/K_A_] Rep 5 | pl1 |  | 7.636 |  | 7.099 | 6.117 | 5.976 | 7.161 |  |
|  | pl2 | 6.086 | 7.714 | 6.498 |  |  |  |  | 6.623 |
| ΔLog[τ/K_A_] Rep 5 | | -1.628 | 0 | -1.216 | -0.537 | -1.519 | -1.66 | -0.475 | -1.091 |
|  |  |  |  |  |  |  |  |  |  |
| **Mean**  **ΔLog[τ/K_A_]** | | **-1.688** |  | **-0.9386** | **-0.4356** | **-1.328** | **-1.644** | **-0.3824** | **-1.179** |
| **SEM** | | **0.051** |  | **0.130** | **0.075** | **0.112** | **0.195** | **0.127** | **0.056** |

| **Nb33** | | **Oxycodone** | **DAMGO** | **Tianeptine** | **Methadone** | **SR-17018**  **mesylate** | **SR-17018** | **Oliceridine** | **Morphine** |
| --- | --- | --- | --- | --- | --- | --- | --- | --- | --- |
| Log[τ/K_A_] Rep 1 | pl1 | 5.63 | 6.758 | 5.621 | 6.15 |  |  |  |  |
|  | pl2 |  | 6.543 |  |  | 4.894 | 5.124 | 5.859 | 5.53 |
| ΔLog[τ/K_A_] Rep 1 | | -1.128 | 0 | -1.137 | -0.608 | -1.649 | -1.419 | -0.684 | -1.013 |
|  |  |  |  |  |  |  |  |  |  |
| Log[τ/K_A_] Rep 2 | pl1 | 5.577 | 7.051 |  |  | 5.471 | 5.547 |  |  |
|  | pl2 |  | 6.879 | 5.518 | 6.475 |  |  | 5.919 | 5.912 |
| ΔLog[τ/K_A_] Rep 2 | | -1.474 | 0 | -1.361 | -0.404 | -1.58 | -1.504 | -0.96 | -0.967 |
|  |  |  |  |  |  |  |  |  |  |
| Log[τ/K_A_] Rep 3 | pl1 |  | 6.289 | 5.686 | 6.188 | 5.02 | 5.175 |  |  |
|  | pl2 | 5.704 | 6.862 |  |  |  |  | 6.225 | 5.916 |
| ΔLog[τ/K_A_] Rep 3 | | -1.158 | 0 | -0.603 | -0.101 | -1.269 | -1.114 | -0.637 | -0.946 |
|  |  |  |  |  |  |  |  |  |  |
| Log[τ/K_A_] Rep 4 | pl1 |  | 6.702 |  | 6.507 | 5.394 | 5.108 | 6.445 |  |
|  | pl2 | 5.513 | 6.933 | 5.574 |  |  |  |  | 5.59 |
| ΔLog[τ/K_A_] Rep 4 | | -1.42 | 0 | -1.359 | -0.195 | -1.308 | -1.594 | -0.257 | -1.343 |
|  |  |  |  |  |  |  |  |  |  |
| Log[τ/K_A_] Rep 5 | pl1 |  | 6.934 |  | 6.603 | 5.32 | 5.86 | 6.669 |  |
|  | pl2 | 5.413 | 6.862 | 5.64 |  |  |  |  | 6.071 |
| ΔLog[τ/K_A_] Rep 5 | | -1.449 | 0 | -1.222 | -0.331 | -1.614 | -1.074 | -0.265 | -0.791 |
|  |  |  |  |  |  |  |  |  |  |
| **Mean**  **ΔLog[τ/K_A_]** | | **-1.326** |  | **-1.136** | **-0.3278** | **-1.484** | **-1.341** | **-0.5606** | **-1.012** |
| **SEM** | | **0.075** |  | **0.14** | **0.088** | **0.081** | **0.105** | **0.134** | **0.091** |

|  | **Oxycodone** | **DAMGO** | **Tianeptine** | **Methadone** | **SR-17018 mesylate** | **SR-17018** | **Oliceridine** | **Morphine** |
| --- | --- | --- | --- | --- | --- | --- | --- | --- |
| **ΔΔLog[τ/K_A_]** | -0.362 |  | 0.1974 | -0.1078 | 0.156 | -0.303 | 0.1782 | -0.167 |
| **Error** | 0.091 |  | 0.191 | 0.115 | 0.138 | 0.221 | 0.185 | 0.106 |
